# Rat hippocampal CA1 region represents learning-related action and reward events with shorter latency than the lateral entorhinal cortex

**DOI:** 10.1038/s42003-023-04958-0

**Published:** 2023-05-31

**Authors:** Shogo Soma, Shinya Ohara, Satoshi Nonomura, Naofumi Suematsu, Junichi Yoshida, Eva Pastalkova, Yutaka Sakai, Ken-Ichiro Tsutsui, Yoshikazu Isomura

**Affiliations:** 1 Brain Science Institute, Tamagawa University, Tokyo, Japan; 2 Department of Molecular Cell Physiology, Kyoto Prefectural University of Medicine, Kyoto, Japan; 3 Laboratory of Systems Neuroscience, Tohoku University Graduate School of Life Sciences, Sendai, Japan; 4 PRESTO, Japan Science and Technology Agency (JST), Kawaguchi, Japan; 5 Department of Physiology and Cell Biology, Graduate School of Medical and Dental Sciences, Tokyo Medical and Dental University, Tokyo, Japan; 6 Center for the Evolutionary Origins of Human Behavior, Kyoto University, Aichi, Japan; 7 Department of Bioengineering, University of Pittsburgh, Pittsburgh, PA USA; 8 Dominick P. Purpura Department of Neuroscience, Albert Einstein College of Medicine, Bronx, NY USA; 9 Department of Clinical Psychology, Pacifica Graduate Institute, Carpinteria, CA USA

**Keywords:** Cortex, Hippocampus, Neural circuits

## Abstract

The hippocampus and entorhinal cortex are deeply involved in learning and memory. However, little is known how ongoing events are processed in the hippocampal-entorhinal circuit. By recording from head-fixed rats during action-reward learning, here we show that the action and reward events are represented differently in the hippocampal CA1 region and lateral entorhinal cortex (LEC). Although diverse task-related activities developed after learning in both CA1 and LEC, phasic activities related to action and reward events differed in the timing of behavioral event representation. CA1 represented action and reward events almost instantaneously, whereas the superficial and deep layers of the LEC showed a delayed representation of the same events. Interestingly, we also found that ramping activity towards spontaneous action was correlated with waiting time in both regions and exceeded that in the motor cortex. Such functional activities observed in the entorhinal-hippocampal circuits may play a crucial role for animals in utilizing ongoing information to dynamically optimize their behaviors.

## Abstract (web summary)

Electrophysiological recordings in rats reveal that the hippocampal CA1 region represents learning-related action and reward events faster than the lateral entorhinal cortex.

## Introduction

The entorhinal cortex (EC) is the major interface between the hippocampus and the neocortex. The EC plays a crucial role, together with the hippocampus, in processing information about ongoing events. Previous anatomical studies in both rodents and primates investigated the hippocampal-entorhinal circuit in detail and showed clear segregation of the hippocampal input and output circuits within the EC layers^1,2^. EC neurons in the superficial layers, namely layer II stellate and fan cells and layer III pyramidal cells, constitute the hippocampal input circuits by projecting to the dentate gyrus (DG) (for layer II cells) and to CA3 and CA1 (for layer III cells)^1,3,4^. The information is processed through the hippocampal trisynaptic circuit (EC layer II → DG → CA3 → CA1), and the direct EC (layer III)-hippocampal input is integrated within CA1 neurons. CA1 is also targeted by the other principal cell type in layer II, pyramidal cells, which have diverse projections to cortical and subcortical structures^4–8^. CA1 neurons, in turn, send the information back to the deep layers of the EC, principally layer V, via the hippocampal output circuit^9–12^ This “entorhinal-hippocampal-entorhinal pathway” is considered to be the main circuit that supports information processing between the hippocampus and neocortex.

In general, CA1 and the EC are involved in spatiotemporal information processing in a distinct manner. For example, the medial EC (MEC) process spatial information in a comprehensive manner through the functions of grid cells^13–15^, border cells^16,17^, head-direction cells^18^, and object-vector cells^19^. Spatial information about the external world is converted into a specific spatial location encoded by place cells in the hippocampus^20–24^. Another part of the EC, the lateral EC (LEC), is intimately involved in processing temporal information. LEC neurons encode elapsed time on a second-to-minute timescale by demonstrating ramping activities related to memory^25^. This temporal information, as well as information from the MEC^26^, can be used to form more specific time-representing activity in the hippocampus (time cells^27,28^). These types of time representations have also been observed in the human LEC and hippocampus^29,30^. Thus, CA1 and the EC have distinct functional roles in the representation of spatiotemporal information.

In addition, the hippocampal-entorhinal circuit is known to represent external event information such as object information^31–33^, sensory/context events^34–40^, episodic-like memory^3,41^, and trace conditioning^42–44^. Furthermore, the hippocampal-entorhinal circuit is involved in associative learning for integrating information from multiple events. For example, CA1 and the LEC increased 20–40 Hz power of local field potentials during odor-association learning, and developed functional activity representing odor information after learning^34^. In fact, optogenetic inhibition of the LEC disrupts sensory cue associative learning^36,37^. Thus, mounting evidence indicates that the hippocampal-entorhinal circuit has a crucial role in associative learning related to various events. In contrast to the distinct representation of spatiotemporal information between CA1 and the EC, little is known about the functional differences in how these areas process event information. The differences in how the superficial and deep EC layers process event information in the entorhinal-hippocampal-entorhinal pathway also remains unknown.

In this study, we investigated the neural representation features of two distinct learning-related behavioral events in the entorhinal-hippocampal-entorhinal pathway, CA1, and superficial and deep LEC layers. By using head-fixed rats that spontaneously manipulated pedals to obtain a reward, we could precisely measure both the elapsed time related to behavioral events and the timing of these events (action and outcome) while monitoring neural activities^45–47^. We extracellularly recorded rat CA1 and LEC neurons both prior to and following training in this task in separate groups of animals. We used these recordings to determine the relationship between behavioral events and spike activities on a sub-second scale. Both CA1 and LEC neurons developed diverse task-related activities after learning, with CA1 representing action and reward events close to real time, and both the superficial and deep layers of the LEC exhibiting much-delayed representation of these events.

## Results

### Self-paced, spontaneous, left or right pedal-releasing task

**Fig. 1:**
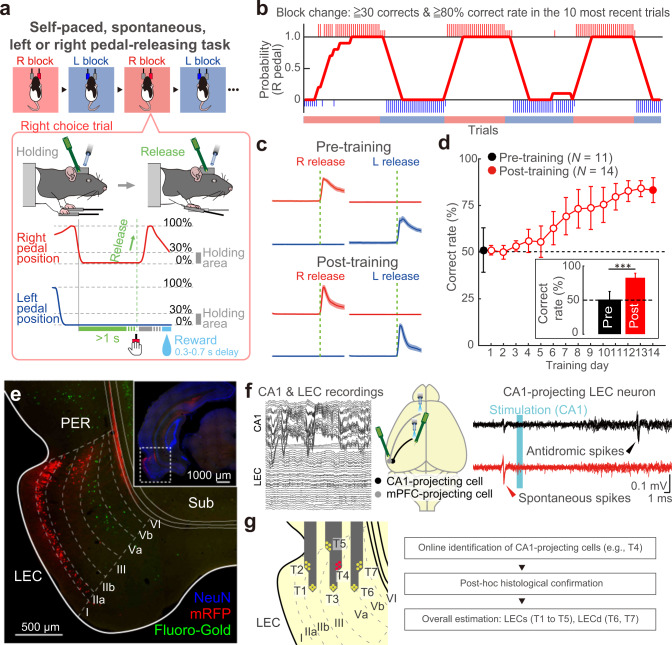
Self-paced, spontaneous, left or right pedal-releasing task and effective LEC recording methods. **a** Schematic diagram of a behavioral task that enabled us to monitor several events with high temporal resolution. A head-fixed rat pushed down both pedals for a short period (≥1 s) to start each trial, and subsequently released either pedal (e.g., right release) voluntarily and without an instruction cue to acquire a reward. The reward was dispensed with a random 300–700 ms delay. This task consisted of right-rewarded (R) and left-rewarded (L) blocks, which were alternated after the rat met the criteria (see Methods). **b** A typical example of task performance. The rat chose the correct pedal based on the reward. Large and small colored vertical bars (red represents right choice; blue represents left choice) indicate correct and incorrect trials, respectively. We averaged the number of right correct choices obtained from the previous 10 trials to calculate the proportion of correct choices. **c** Right-left pedal trajectories obtained from pre-trained (1^st^ day, top) and post-trained (14^th^ day, bottom) rats. **d** Learning curve over 14 training days. Inset, averaged proportion of correct choices on the 1^st^ and 14^th^ days. Black and red colors represent the pre- and post-training groups, respectively. ***p < 0.001, Mann–Whitney test. Error bars indicate SD. **e** LEC neurons projecting to the mPFC and hippocampus. Retrograde tracer (Fluoro-Gold) was injected into the mPFC, while retrograde viral tracer (mRFP-expressing G-deleted rabies viral vector) was injected into the hippocampus (Supplementary Fig. 2a). The distribution of retrogradely labeled neurons was subsequently examined in the LEC. Note that mPFC-projecting neurons (green) are found in layer Va while hippocampus-projecting neurons (red) are found in layers IIa and III of the LEC. **f** Example traces of simultaneous CA1 and LEC recording (left). Schema showing the position of optical fibers for identifying the two different projection neurons in the LEC (middle). The ipsilateral mPFC and CA1 were stimulated to identify mPFC- and CA1-projecting LEC neurons, respectively (Supplementary Fig. 2b). Example of recordings from a single CA1-projecting LEC neuron during optical stimulation (cyan area), with spike collisions. Black and red traces represent antidromic spikes in response to optical stimulation and spike collision tests, respectively. Black arrowheads indicate antidromic spikes. Red arrowheads indicate spontaneous spikes used as triggers for optical stimulation in collision tests (right). **g** The laminar position of the recording site for LEC cells was reconstructed using online and offline estimations. While recording, the recording depth was quickly determined by identifying CA1-projecting LEC neurons (**f**). After recording, probe shank tracks were visualized with DiI (Supplementary Fig. 2b).

In order to precisely monitor and measure the timing of behavioral events, we adopted a simple behavioral task: a self-paced, spontaneous, left or right pedal-releasing task (Fig. 1). The rats used in our study had to manipulate left and right pedals with the corresponding forelimb in a head-fixed condition, which enabled us to monitor the accurate timing of events (action and outcome). The rats started each trial spontaneously by pushing both pedals down with the left and right forelimbs and holding them for a constant period in a self-paced manner. The rats had to choose to release either the left or right pedal (action) without any instruction cue to obtain saccharin water as a reward (outcome). The task consisted of two blocks (right pedal–rewarded and left pedal–rewarded), and the reward pedal was changed in a block-by-block manner with no instruction (R, R, R, R… L, L, L, L…; Fig. 1a, b).

Figure 1c show the pedal traces on the left- or right-releasing trials obtained from pre- and post-training groups. Both groups could manipulate the individual pedals spontaneously (see also Supplementary Fig. 1a). Thus, rats could manipulate the pedal i.e., they could spontaneously express the motor response (unilateral pedal release), before they learned the task rule. In contrast, other measures such as holding stability and release time (time from onset to end of release) changed during the course of training. Rats in the post-training group quickly released the pedal and returned their forelimb to the pedal after stable pedal holding (Supplementary Fig. 1b), indicating that the rats learned the precise motor response (skilled pedal manipulation) over the course of training.

The rats typically learned this task within 2 weeks (Fig. 1d). The most remarkable difference between groups was in task performance. Rats in the pre-training group manipulated the pedals randomly whereas those in the post-training group chose the appropriate pedals and manipulated them based on the task rule (Mann–Whitney test, *z* = 4.13, *p* < 3.6 × 10^−5^, *r* = 0.83; Fig. 1d, Supplementary Fig. 1c and Supplementary Data 1).

### Recording and cell-type classification in the hippocampal formation

We recorded multineuronal spike activity (i.e., multiple, isolated single units) by inserting silicon probes into the hippocampal formation (CA1 and LEC) of rats that performed the self-paced, spontaneous, left or right pedal-releasing task. Based on the dense connectivity between the LEC and distal CA1, we aimed to begin recording in the distal half of the CA1 region^48^. We first verified the connectivity patterns of the LEC using two retrograde tracers, Fluoro-Gold and G-deleted rabies viral vector (ΔG-RV)^49^ expressing monomeric red fluorescent protein (mRFP). In line with previous studies^1,11^, injection of Fluoro-Gold into the medial prefrontal cortex (mPFC) resulted in labeling of neurons in layer Va, while ΔG-RV injection into the DG and CA1 led to mRFP labeling neurons mainly in layers IIa and III of the LEC (Fig. 1e, Supplementary Fig. 2a). In this study, we focused on mPFC- and CA1-projecting LEC neurons that are found mostly in layers Va and III, respectively, as this enabled us to reconstruct the layer position of recorded neurons with increased certainty. We identified these neuron classes using the Multi-Linc (multi-areal/multineuronal light-induced collision) method^45,46,50–52^, with antidromic stimulation of the ipsilateral mPFC for mPFC-projecting neurons and of the ipsilateral CA1 for CA1-projecting neurons (Fig. 1f). We reconstructed the recording and stimulating sites histologically after recording (Supplementary Fig. 2b).

The right panel of Fig. 1f shows typical traces of antidromic spikes (black) and their disappearance due to collisions with spontaneous spikes (red) in a single CA1-projecting LEC neuron. Since LEC neurons near the rhinal fissure project into the dorsal CA1 (Fig. 1e), we could verify that our recording electrode was located in the LEC based on the presence of such CA1-projecting neurons (Fig. 1f), resulting in effective in vivo LEC recording of behaving rats (Supplementary Figs. 2 and 3). We monitored the CA1-projecting neurons online and determined the probe position that would record the maximum number of neurons in every recording session (number of identified cells/session offline, pre-training: LEC → mPFC cells, 0.64 ± 1.0 (mean ± SD), LEC → CA1 cells, 1.3 ± 1.9; post-training: LEC → mPFC cells, 2.4 ± 4.9, LEC → CA1 cells, 2.5 ± 4.5). We compared the antidromic latency between CA1- and mPFC-projecting neurons and found no difference (pre-training: LEC → mPFC cells, *n* = 7, median [IQR] in ms, 17.1 [12.3, 19.2], LEC → CA1 cells, *n* = 16, 19.7 [14.5, 22.2]; Mann–Whitney test, *z* = −1.44, *p* = 0.15, *r* = 0.30; post-training: LEC → mPFC cells, *n* = 25, 21.7 [16.7, 26.7], LEC → CA1 cells, *n* = 34, 22.3[17.7, 25.2]; *z* = 0.00, *p* = 0.99, *r* = 0.00; Supplementary Fig. 2c). There was also no difference in the antidromic latency between pre- and post-training (LEC → CA1 cells, *z* = 1.84, *p* = 0.07, *r* = 0.28; LEC → mPFC cells, *z* = 1.15, *p* = 0.25, *r* = 0.18).

Our multineuronal recordings during task performance in the pre- and post-training groups isolated 829 CA1 neurons and 1287 LEC neurons, the majority of which were putatively classified (see Methods for details). Since LEC neurons in the superficial and deep layers send their projections to different brain regions^1^, we grouped the putative superficial layer (LECs) cells and putative deep layer (LECd) cells based on the results of optogenetic identifications and histological observations (Fig. 1g). All recorded neurons were further classified as either regular-spiking (RS, mostly putative excitatory neurons) or fast-spiking (FS, putative inhibitory neurons) neurons based on minimum cross-entropy thresholding of spike duration (Supplementary Fig. 3). Consistent with previous reports^53,54^, the ongoing spike rates of FS subtypes were significantly higher than those of RS subtypes in CA1 and the LEC (Supplementary Fig. 3). Given the small sample size of task-related FS neurons, we used RS neurons in further analyses.

### Development of task-related activities in CA1 and LEC cells

**Fig. 2:**
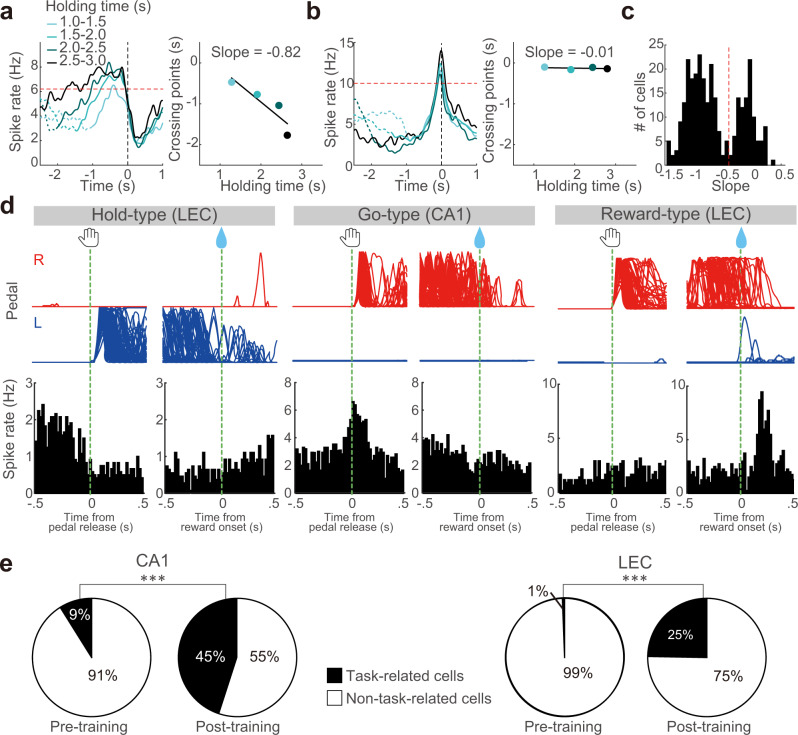
Task-related activities developed dramatically during learning. Classification of Hold-type (**a**) and Go-type (**b**) activities according to the dependence of spike increase on behavioral holding time. Left, PETHs from a representative LEC neuron calculated from trials with varying ranges of holding time (light to dark blue). Right, plot of the intersection with criterion (red dashed line, 75% of peak activity in all-averaged PETH) against the four ranges of holding time, in which we obtained the slope value from linear regression. The slope was negative for Hold-type activities (**a**) and near zero for Go-type activities (**b**). **c** Clear bimodal distribution of slope values in all task-related neurons with their peak time before the pedal release. Red dashed line indicates threshold for classification into Hold-type (toward negative) and Go-type (near zero). **d** Examples of Hold-type (left), Go-type (middle), and Reward-type (right) task-related activities in CA1 (middle) and the LEC (left, right) after learning. Top and bottom show pedal trajectories (red: right pedal, blue: left pedal) and PETHs (bin width, 20 ms), respectively. Spike data are aligned with the pedal release onset (left) or reward onset (right) at 0 s for individual task-related neurons. **e** Population ratios of task-related neurons in CA1 and the LEC of the pre- and post-training groups. ***p < 0.001, 2 ×2 χ^2^ test.

We first compared the ongoing spike rate between the pre- and post-training groups, and found a significantly reduced spike rate in CA1 and the LEC after learning (CA1-RS: pre-training, *n* = 247, median [IQR] in Hz, 3.2 [1.8, 6.1], post-training: *n* = 433, 2.8 [1.1, 5.3], Mann–Whitney test, *z* = −2.76, *p* < 5.2 × 10^−3^, *r* = 0.10; LEC-RS: pre-training, *n* = 335, 1.6 [1.1, 2.4], post-training, *n* = 789, 0.8 [0.4, 1.4], *z* = −13.2, *p* < 7.5 × 10^−40^, *r* = 0.39; see also Supplementary Fig. 3). This suggests that training resulted in neuronal changes in the hippocampal-entorhinal circuit. Therefore, we next examined if CA1 and LEC neurons correlated to the task events, and observed various types of task-related neurons in CA1 and the LEC of rats performing the self-paced forelimb pedal-releasing task. We classified task-related neurons as Hold-type, Hold&Reward-type, Go-type, Go&Reward-type, and Reward-type according to their preferred activities, namely contralateral or ipsilateral activities (Fig. 2, Supplementary Fig. 4; see Methods), with task relevance indices based on previous studies^45–47^. Briefly, we observed both holding time-dependent and -independent activities, i.e., Hold-type and Go-type activities, respectively (Fig. 2a, b). The peri-event time histograms (PETHs) of Hold-type neurons were characterized by ramping or sustained activity during the holding period (Fig. 2a). In contrast, Go-type neurons exhibited peak phasic activity just before pedal release, independent of holding time (Fig. 2b). We observed a bimodal distribution in the dependency of spike activity on holding time, clearly dividing the Hold-type and Go-type neurons (threshold at 0.5 in slope; Fig. 2c).

**Fig. 3:**
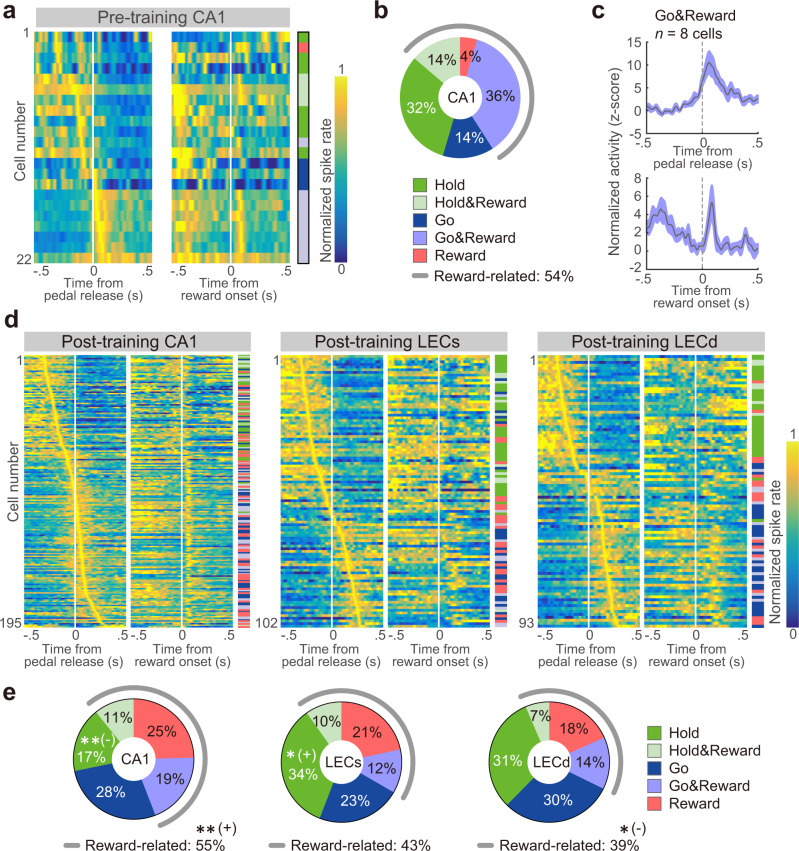
The repertoire of task-related activities in CA1 neurons before and after learning. **a** Five types of task-related activities in the RS subtype of CA1 neurons before learning. Normalized Gaussian-filtered PETHs (σ = 12.5 ms for spikes in 0.05 ms bins) aligned with pedal-release onset and reward onset at 0 s (vertical line) for individual task-related neurons. Each row represents a single neuron; they were sorted by the order of peak time obtained from pedal-release onset data (early to late). The task-related type is indicated on the right side. **b** Population ratios of task-related types in CA1-RS neurons. **c** Averaged PETHs of all Go&Reward-type activities in CA1 of the pre-training group. PETHs were aligned with pedal-release onset (top) and reward onset at 0 s (bottom). Shaded regions represent 95% confidence intervals (CIs). **d** Five types of task-related activities in the RS subtypes of CA1 and the LEC after learning. The figure legend is the same as in **a**. **e** Population ratios of task-related types in the RS neurons of CA1 and the LEC after learning. The figure legend is the same as in **b**. **p < 0.01, Residual analysis after a 5 ×2 χ^2^ test.

**Fig. 4:**
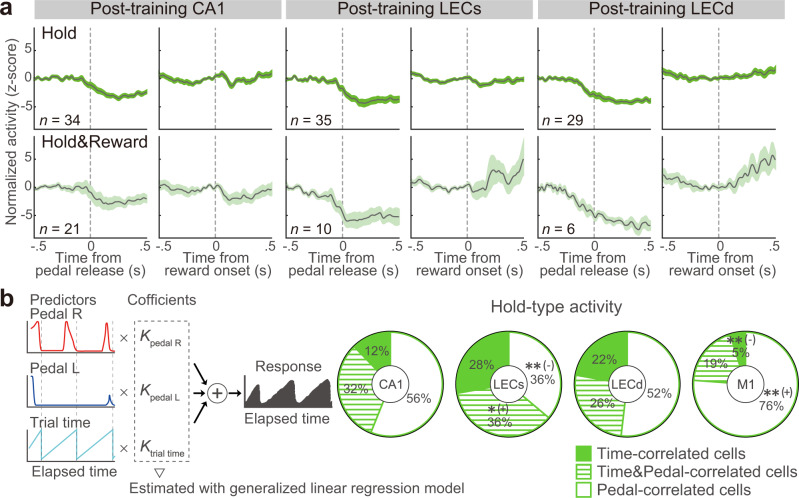
Holding time representation of ramping activities. **a** Averaged PETHs of all Hold- and Hold&Reward-type activities in CA1 (left), LECs (middle), and LECd (right). PETHs were aligned with pedal-release onset (left columns) and reward onset at 0 s (right columns). Shaded regions represent 95% CIs. **b** Schematic of a generalized linear regression model used to determine if each Hold-type cell represents the pedal trajectories, trial time, or both. Coefficients for relevant predictors were determined with a stepwise selection process to estimate smoothed spiking activities with a summation of weighted temporal traces of the left and right pedal trajectories and elapsed time in a trial (between two sequential pedal-holding onsets) (left). *P*-values with Bonferroni correction of each coefficient were used to classify cells as Time-, Time&Pedal-, or Pedal-correlated cells. Proportion of Time-, Time&Pedal-, and Pedal-correlated cells for CA1, LECs, LECd, and M1 (right).

**Fig. 5:**
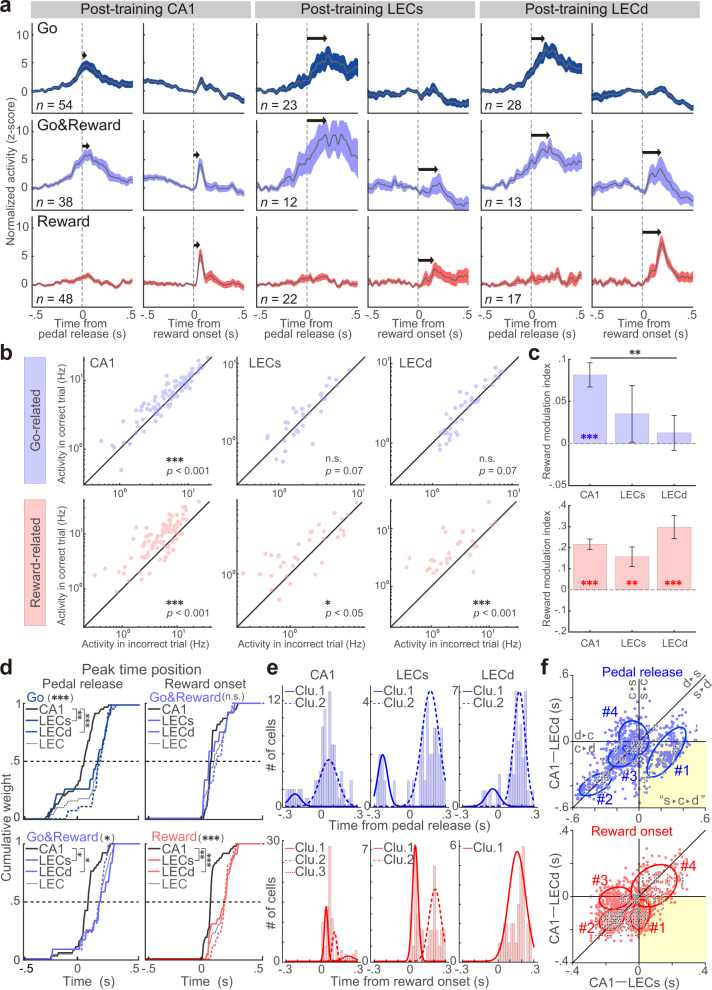
Action and outcome representations of phasic activities. **a** Averaged PETHs of all Go-, Go&Reward-, Reward-type activities in CA1 (left), LECs (middle), and LECd (right). The figure legend is the same as in Fig. 4a. Arrows show the peak time. **b** Reward modulation of neuronal activity. The spike rates of go- (top) and reward- (bottom) related neurons obtained from correct and incorrect trials are plotted. **c** Comparison of reward modulation index. **p < 0.01, post hoc Steel–Dwass test. Colored asterisks indicate significant positive reward modulation. **p < 0.01, ***p < 0.001, one-sample signed-rank test. Error bars indicate SEM. **d** Cumulative distributions of peak time position from the onset of pedal release (left; Go- (top) and Go&Reward-type (bottom)) and from the onset of reward delivery (right; Go&Reward- (top) and Reward-type (bottom)). *p < 0.05, **p < 0.01, ***p < 0.001, post hoc Steel–Dwass test. **e** Distribution of peak time positions from the onset of pedal release (top) and from the onset of reward delivery (bottom) in CA1 (left) as well as LECs (middle) and LECd (right). The number of clusters was determined based on BIC for the results of GMM fitting with the EM algorithm (see Supplementary Fig. 7a). **f** A bootstrap analysis (1000 samples) was performed on the data to visualize possible information flows (colored dots). The number of clusters was determined based on the BIC for the results of the 2D GMM fitting with the EM algorithm (see Methods). Horizontal, vertical, and diagonal lines indicate the borders of firing orders of the three regions (see appended annotations). A pale yellow background indicates a quadrant corresponding to what one would expect based on the LECs → CA1 → LECd circuit. c, CA1; s, LECs; d, LECd. Black dots show representative data obtained from a single session.

Figure 2d shows the PETHs of representative neurons involved in pedal holding (Hold-type in the LEC, left), pedal release (Go-type in CA1, middle), and reward delivery and/or consumption (Reward-type in the LEC, right; see also Figs. 3–5, Supplementary Figs. 4–6). In the pre-training group, there were few task-related cells in CA1 and the LEC. In contrast, the fractions of task-related cells in both CA1 and the LEC were dramatically increased after rats learned the task rule (CA1-RS; χ^2^ test, χ^2^ = 94.5, *p* < 2.5 × 10^−22^, *φ* = 0.37; LEC-RS: *n* = 594; χ^2^ = 94.6, *p* < 2.3 × 10^−22^, *φ* = 0.53; Fig. 2e and Supplementary Data 1).

In the pre-training group, CA1 neurons included five types of task-related neurons (Fig. 3a, b and Supplementary Data 1). We found that before task learning, the dominant fraction of task-related neurons represented both pedal release and reward (Go&Reward-type) (Fig. 3b, c). In contrast, only two LEC neurons represented task-related activity (Supplementary Data 1).

Next, we determined the types of task-related activity that developed after learning in CA1 and the LEC (Fig. 3d, e). After learning, all five types of task-related activities were observed in both CA1 and the LEC (Fig. 3d and Supplementary Data 1). LECs and CA1 had significantly larger and smaller populations of Hold-type neurons, respectively (χ^2^ test, χ^2^ = 15.7, *p* < 4.7 × 10^−2^, *φ* = 0.20; post hoc residual analysis: CA1, *p* < 0.05; LECs *p* < 0.01; Fig. 3e).

Since the outcome-related activities seemed more predominant in CA1 compared to the LEC, we conducted further analyses after pooling the reward-related activity types as the Reward-related type (CA1-RS: *n* = 107 (54.9%), LECs-RS: *n* = 44 (43.1%), LECd-RS: *n* = 36 (38.7%); Fig. 3e). As expected, outcome-related information was predominantly encoded by CA1 (χ^2^ test, χ^2^ = 7.9, *p* < 2.0 × 10^−2^, *φ* = 0.14; post hoc residual analysis, *p* < 0.01). Also, the LECd showed a significantly smaller population of outcome-related neurons than others (*p* < 0.05). Thus, both CA1 and LEC neurons exhibited a varied repertoire of task-related activities after learning.

We also examined task-related activities of identified projection LEC neurons (Supplementary Fig. 5). Supplementary Fig. 5a is a representative example of an mPFC-projecting LEC neuron that showed Go-type activity, which suggests that this neuron sends action-related information to the mPFC. Supplementary Fig. 5b shows the task-related activities of all identified neurons. Consistent with the current findings (Supplementary Fig. 5) and previous histological observations^11^, the mPFC- and CA1-projecting LEC neurons were recorded from deep and superficial layers, respectively. The only exception was mPFC-projecting LEC neurons in the superficial layer, which likely corresponded to calbindin-positive neurons in LEC layer IIb^8^. Both projection neurons showed action- and outcome-related activities. The outcome-related activities were often observed in CA1-projecting LEC neurons rather than mPFC-projecting neurons (LEC → dCA1: 6/10 cells (60%); LEC → mPFC: 3/9 cells (33.3%)). Although this tendency was not tested for statistical significance because of the small sample size, we obtained a similar result by comparing reward-related fractions between the LECd and LECs (Fig. 3e). Thus, like unidentified neurons, identified projection neurons also showed a varied repertoire of task-related activities after learning.

### Spike rate and limb specificity of action- and outcome-related CA1 and LEC neurons after learning

We compared the spike rates associated with hold-related activities (Hold- and Hold&Reward-type) by averaging the spike activities during the pre-movement period (−1000 to −500 ms). The LECd showed a significantly lower spike rate than both CA1 and the LECs (Supplementary Fig. 6a, b, top). We next calculated the peaks ( ± 150 ms) for action-related (Go- and Go&Reward-type) and outcome-related (Reward- and Go&Reward-type) activities. For the action-related types, CA1 showed significantly higher spiking activities than both the LECs and LECd (Supplementary Fig. 6a, b, middle). Additionally, the peaks for outcome-related activities in CA1 were significantly higher than those in LEC. In addition, we observed a significant difference in peak activities between the LECs and LECd (Supplementary Fig. 6a, b, bottom). Thus, we found contrasting peak activities in superficial versus deep LEC layers.

Since our original task can evaluate laterality^45–47^, i.e. the preference for contra- or ipsilateral limb movement of a task-related activity, we tested if CA1 and LEC neurons showed lateralized activity. For action-related activities (Go- and Go&Reward-types), Go-type CA1 neurons preferred ipsilateral activity but other subpopulations did not show lateralized activities (Supplementary Table 1). Thus, both CA1 and LEC neurons have basically bilateral activity, and the ipsilateral preference of Go-type CA1 neurons is similar to that of the posterior parietal cortex (PPC)^47^. For outcome-related activities (Go&Reward- and Reward-types), neither CA1 nor LEC showed evidence of laterality (Supplementary Table 2).

### Holding-time representation with ramping activities of CA1 and LEC neurons

Hold-type neurons showed sustained-like activities that ramped up prior to the pedal release (Figs. 2–4). Since CA1 and LEC neurons are known to represent time^25,27^ differently from neurons in the primary motor cortex (M1), which represent the pedal holding purely as a motor command^46^, we further investigated the functional significance of Hold-type neurons. Specifically, we sought to determine if single CA1 and LEC neurons represent the holding action or the time elapsed while holding the pedal. If neurons represent the holding action, their activities should be more correlated to pedal traces than elapsed time. In contrast, if neurons represent the holding time, their activities should be more correlated to elapsed time than pedal traces. We performed generalized linear regression analysis by using the pedal traces (e.g., spatial positions of pedals, speed of pedal manipulation, small variances during pedal holding, etc.) and holding time (self-started trial time: typically, 1–3 s; see Fig. 2a) as predictors (Fig. 4b). As expected, the M1 neurons predominantly represented pedal traces (Pedal-correlated cells), in contrast with both CA1 and LEC neurons that represented holding time on a sub-second scale (Time- and Time&Pedal-correlated cells) in particular, the LECs showed a relatively large proportion of time-related cell types than the other areas (CA1: 44%, LECs: 64%, LECd: 48%, M1: 24%; Supplementary Data 1). This tendency was confirmed to be statistically significant (χ^2^ test, χ^2^ = 15.5, *p* < 1.6 × 10^−2^, *φ* = 0.33; post hoc residual analysis, *p* < 0.05). Since the LECs also showed a significantly larger Hold-type population than the other areas (Fig. 3e), the LECs might have an important role in initially representing the holding time, which can subsequently be used as a signal to initiate voluntary action.

### Temporal dynamics of action- and outcome-related CA1 and LEC neurons after learning

To visualize the temporal dynamics of other types of neurons, we calculated averaged PETHs (Fig. 5a). In contrast to Hold- and Hold&Reward-type neurons, Go-, Go&Reward-, and Reward-type neurons showed clear peaks in both CA1 and LEC. One of the most interesting differences in the average activity patterns involved the post-reward responses in CA1 versus LEC (Fig. 5a and Supplementary Fig. 6). Since our task enabled us to evaluate the reward modulation by comparing the activities between correct and incorrect trials^46^, we investigated if the activities of go- and reward-related neurons were modulated by the presence or absence of a reward (Fig. 5b, c and Supplementary Data 1). Most data points of reward-related neurons fell above the diagonal line, showing that their activities were increased by a reward (Wilcoxon signed-rank test, CA1: *p* < 6.2 × 10^−11^, *z* = 6.54, *r* = 0.71; LECs: *p* < 1.6 × 10^−2^, *z* = 2.41, *r* = 0.41; LECd: *p* < 2.8 × 10^−4^, *z* = 2.41, *r* = 0.44; Fig. 5b, bottom). This was further confirmed by positive values for the reward modulation index (one-sample signed-rank test, CA1: *p* < 1.1 × 10^−10^, *z* = 6.45, *r* = 0.70; LECs: *p* < 2.9 × 10^−3^, *z* = 2.98, *r* = 0.50, LECd: *p* < 6.9 × 10^−5^, *z* = 3.98, *r* = 0.73; Fig. 5c, bottom). There was no significant difference in the reward modulation index between CA1, LECs, and LECd (Kruskal–Wallis test, χ^2^ = 0.4, *p* = 0.09, *η*^*2*^ = 0.03). Surprisingly, the go-related neurons in CA1 but not the LEC showed positive reward modulation during correct trials (Wilcoxon signed-rank test, CA1: *p* < 1.2 × 10^−7^, *z* = 5.30, *r* = 0.55; LECs: *p* = 0.07, *z* = 1.81, *r* = 0.31; LECd: *p* = 0.07, *z* = 1.80, *r* = 0.28; Fig. 5b, top; one-sample signed-rank test, CA1: *p* < 3.1 × 10^−3^, *z* = 5.12, *r* = 0.53; LECs: *p* = 0.24, *z* = 1.16, *r* = 0.20, LECd: *p* = 0.56, *z* = 0.58, *r* = 0.09; Kruskal–Wallis test, χ^2^ = 6.7, *p* < 3.6 × 10^−2^, *η*^*2*^ = 0.04; post hoc Steel–Dwass test, CA1 vs. LECd, *p* < 0.01; Fig. 5c, top). This positive modulation by the reward was similar to the reward modulation previously observed in the motor cortices and striatum^46,55–57^.

Another interesting observation in the average activity patterns was that CA1 neurons had a shorter peak latency than LEC neurons at the population level (arrows; Fig. 5a). To quantify this observation, we calculated peak latency for both CA1 and LEC (Fig. 5d and Supplementary Data 1). First, we compared the peak latency of Go- and Go&Reward-types obtained from the PETHs aligned with pedal release, and found that CA1 had a significantly shorter latency than the LEC for both activity types (Go-type: median [IQR] in ms, CA1, 31.8 [−60.0, 82.0]; LECs, 147.0 [−84.25, 212.9]; LECd, 166.8 [121.0, 217.0], Kruskal–Wallis test, χ^2^ = 29.7, *p* < 3.6 × 10^−7^, *η*^*2*^ = 0.23; post hoc Steel–Dwass test, CA1 vs. LECs, *p* < 3.6 × 10^−7^, *t* = 2.95, Cliff’s *d* = 0.43; CA1 vs. LECd, *p* < 6.1 × 10^−7^, *t* = 5.41, Cliff’s *d* = 0.73; LECs vs. LECd, *p* = 0.15, *t* = 1.04, Cliff’s *d* = 0.17; Fig. 5d, top; Go&Reward-type: CA1, 61.0 [17.0, 84.0]; LECs, 161.3 [74.8, 245.0]; LECd, 158.0 [75.6, 191.0]; χ^2^ = 8.4, *p* < 1.5 × 10^−2^, *η*^*2*^ = 0.12; CA1 vs. LECs, *p* < 1.2 × 10^−2^, *t* = 2.39, Cliff’s *d* = 0.46; CA1 vs. LECd, *p* < 1.9 × 10^−2^, *t* = 2.13, Cliff’s *d* = 0.40; LECs vs. LECd, *p* = 0.23, *t* = 0.70, Cliff’s *d* = −0.17; Fig. 5d, bottom). We also compared this parameter for Reward- and Go&Reward-types obtained from PETHs aligned with reward delivery. For the Go&Reward-type, there was no significant difference in peak latency between the three groups (Go&Reward-type: CA1, 70.3 [48.5, 85.0]; LECs, 62.5 [30.8, 173.3]; LECd, 124.0 [53.5, 189.9]; χ^2^ = 2.1, *p* = 0.33, *η*^*2*^ = 0.04; CA1 vs. LECs, *p* = 0.23, *t* = 0.80, Cliff’s *d* = −0.15; CA1 vs. LECd, *p* = 0.18, *t* = 0.98, Cliff’s *d* = 0.23; LECs vs. LECd, *p* = 0.09, *t* = 1.31, Cliff’s *d* = 0.27; Fig. 5d, top). Conversely, Reward-type CA1 neurons showed a significantly shorter peak latency than LEC neurons in both superficial and deep layers (Reward-type: CA1, 69.0 [58.3, 84.5]; LECs, 192.3 [62.0, 233.0]; LECd, 195.5 [153.1, 211.5]; Kruskal–Wallis test, χ^2^ = 2.2, *p* = 0.34, *η*^*2*^ = 0.04; post hoc Steel–Dwass test, CA1 vs. LECs, *p* = 0.23, *t* = 0.80, Cliff’s *d* = −0.15; CA1 vs. LECd, *p* = 0.09, *t* = 1.32, Cliff’s *d* = 0.25; LECs vs. LECd, *p* = 0.18, *t* = 0.98, Cliff’s *d* = 0.23; Fig. 5d, bottom).

The shape of the cumulative curves seems to comprise several ramps, which suggests the presence of subpopulations with different peak time positions. We tested this point by fitting a peak latency histogram to a Gaussian mixture model (GMM; Fig. 5e). Cluster numbers were determined based on the Bayesian information criterion (BIC; Supplementary Fig. 7a). For action-related activity types (Go- and Go&Reward-types), all three regions showed two distinct subpopulations: those preceding and those following spike activity relative to pedal release (CA1, median [IQR] in ms, −224.7 [−257.5, −191.9] and 62.2 [11.5, 112.9]; LECs, −227.4 [−254.9, −199.9] and 174.8 [125.7, 224.0]; LECd, −48.0 [−84.9, −11.0] and 177.0 [143.9, 210.0]; Fig. 5e, top). We verified this result with another method, *x*-means clustering (Supplementary Fig. 7b, top). For outcome-related activity types, the latencies relative to the reward onset timing were clustered into multiple groups in some regions (CA1 = three clusters, 39.2 [29.2, 49.2], 110.0 [−97.8, 122.2], and 226.7 [192.8, 260.6]; LECs = two clusters; 47.3 [31.3, 63.3] and 214.2 [179.9, 248.5]; LECd = one cluster, 158.7 [111.3, 206.1]; Fig. 5e, bottom), although *x*-means clustering showed that all three regions had two distinct subpopulations (Supplementary Fig. 7b, bottom). These results led us to speculate that distinct subpopulations send their signals to other populations with different timings.

To visualize pseudo-signal flow between the three regions, we calculated the pseudo-paired differences of peak latency between neurons in different pairs of regions (e.g., CA1 vs. LECs, and CA1 vs. LECd; Fig. 5f, BIC; Supplementary Fig. 7c; see Methods). We found that action-related neurons included the subpopulation reflecting the hippocampal-entorhinal circuit (cluster #1 in the pale-yellow background, LECs → CA1 → LECd; Fig. 5f, top), and other clusters (#2 and 3) showed that the CA1 neurons act before LEC neurons. The outcome-related neurons did not include a subpopulation reflecting the hippocampal-entorhinal circuit, but other subpopulations instead; this showed that CA1 and LECs neurons act simultaneously and send their signals to LECd neurons (cluster #1; Fig. 5f, bottom). As shown in Fig. 5f, cluster #2 showed that CA1 neurons act prior to LEC neurons. A similar tendency was observed in the single-session data (black dots). These results suggest the existence of subpopulations of CA1 and LEC neurons that process information in a different order from that suggested by previous anatomical findings, i.e., the hippocampal-entorhinal circuit.

## Discussion

To investigate the characteristics of neural representation for two distinct behavioral events related to learning in the hippocampal-entorhinal circuit, we recorded neuronal activities in CA1 and the LEC (superficial and deep layers) while rats performed a simple behavioral task requiring a spontaneous action of pedal release to acquire a reward. Our main findings are as follows: (1) the proportion of task-related neurons that showed task-related activity increased in both CA1 and the LEC after learning; (2) five types of task-related activities (Go-type, Go&Reward-type, Hold-type, Hold&Reward-type, and Reward-type) were observed, and the LEC developed a larger population of the Hold-type than CA1; (3) both CA1 and the LEC represent the holding time on a sub-second scale; (4) reward-related neurons in both CA1 and the LEC showed facilitated reward modulation during reward delivery, while only CA1 go-related neurons showed positive reward modulation during correct trials prior to reward delivery; (5) peak latency was shorter for CA1 than the LEC among Go-type, Go&Reward-type, and Reward-type activities; (6) each area contained distinct clusters showing different peak time positions, and action- but not outcome-related neurons included subpopulations reflecting the hippocampal-entorhinal circuit; and (7) the mPFC- and CA1-projecting LEC neurons identified with Multi-Linc represented both action- and outcome-related information.

Both CA1 and LEC neurons exhibited task-related activities after learning. These activities in the hippocampal-entorhinal circuit were thought to be neuronal representations acquired while animals experienced various events during task learning. Before the rats learned the task rule, only a few LEC neurons showed task-related activities (<1%); however, a substantial number of CA1 neurons responded to behavioral events (9%; Fig. 2e). These CA1 neurons mainly consisted of the Go&Reward-type (Fig. 3a–c), which suggests that there are CA1 neurons (undifferentiated neurons) sensitive to multiple events in the early phase of learning. Moreover, these neurons contribute to the formation of action-outcome contingency through behavioral events. In contrast, task-related LEC activity newly appeared after learning (Fig. 2e). LEC neurons can represent different types of information after learning^31–39,58^. These distinctions between CA1 and LEC neurons suggest that CA1 acts as a foundation for providing ongoing information about task learning to the LEC in the early phase of learning, so that the LEC can adapt to handle this information as learning progresses (cf. ref. ^34^). In addition to the go- and reward-related types, neurons that exhibited hold-related activities appeared after learning, indicating that these activities were not simple waiting activities as previously observed in the motor cortex^46^ but task-relevant ones that may represent the time to express the specific behavior involved in task learning. In fact, Hold-type neurons representing the holding time were mostly distributed in the LEC (Figs. 3d, e and 4*; see below for further discussion*), which is consistent with a previous study in which relatively longer time representation was found in the LEC^25^.

The hippocampal-entorhinal circuit is important for time representation^25–30^. For example, both deep and superficial layers of LEC neurons represent time over timescales of minutes or longer^25^. In addition to this macroscopic time representation of experiences, we observed a sub-second-scale time representation (Fig. 4). Since the rats were head-fixed and our task did not include an instruction cue, the rats had to measure the trial time internally without moving. This holding time representation was largely observed in the LECs (Fig. 3e), which is where the entorhinal-hippocampal-entorhinal pathway begins^1,48^, suggesting that the LECs has an important role in representing holding time information and thus initiating voluntary action (see Hold-related LECs neurons in Supplementary Fig. 5). To determine if the LECs plays such a role, in the future we need to efficiently identify a large number of LECs neurons using a cutting-edge cell identification method such as automated, parallelized Multi-Linc analysis^59^.

Both CA1 and LEC neurons showed an essentially bilateral preference, although Go-type CA1 neurons had a subtle ipsilateral bias (Supplementary Table 1). These results indicate that go-related activity in CA1 and the LEC could represent abstract information for using motion expression. Similarly, previous studies have demonstrated that voluntary forelimb movement is bilaterally or slightly ipsilaterally biased in the PPC, and the PPC processes abstract information, whereas the primary motor cortex shows contralateral representation and is involved in concrete motor information^46,47^. The peak time position of action-related neuron types (Go- and Go&Reward-types) was shorter in the CA1 than in the LEC. Our analysis revealed two subpopulations in CA1 and the LEC, defined according to the timing of spike activity: preceding or following pedal release (Fig. 5). The former neurons are thought to be involved in preparation or planning for voluntary movement, while the latter may exert feedback activities that regulate or monitor expressing movements. In fact, the LEC directly received inputs from both the PPC and motor cortices^60,61^. Visualization of pseudo-signal flow suggested that task-related information was processed though distinct subpopulations: LECs → CA1 → LECd or CA1 → LECs & LECd (Fig. 5f, top). Thus, it was revealed that CA1 and some LECs neurons first represent the preparation or planning for voluntary expressing movement. Next, most of the remaining CA1 neurons act instantaneously, and the LECs and LECd neurons act in succession during actual movement in the hippocampal-entorhinal circuit.

Based on anatomical studies, sensory information about the auditory signal for reward presentation (audition) and licking (somatosensation) is expected to be processed sequentially through the entorhinal-hippocampal-entorhinal pathway: LECs → CA1 → LECd. In contrast to the canonical sequence for information processing, the reward-responsive activities of CA1 neurons (Reward-type and Go&Reward-type) preceded those of LEC neurons, and CA1 showed a sharp peak compared to the LEC (Fig. 5). Since licking continues for a few seconds, it is unlikely that LEC neurons represent drinking behavior with licking. In the pre-training group, Reward-type neurons were rare in both CA1 and the LEC (Figs. 2 and 3). Therefore, it is likely that the Reward-type activity of CA1 and LEC neurons does not comprise simple sensory responses but rather learning-related activities that have developed through task training.

A similar inconsistency between connectivity and activity patterns was reported by a study that examined the processing of olfactory sensory inputs using an in vitro–isolated guinea pig brain preparation^62^. The neural activity induced by the lateral olfactory tract stimulation propagated sequentially from the LEC to the hippocampus, and from the hippocampus to the MEC but not the LEC. This finding, together with our results, implies that information processing through the entorhinal-hippocampal-entorhinal pathway is more complex than initially reported. One possible explanation for early reward representation in CA1 is the direct input of reward signals to CA1 that bypasses the EC. Indeed, CA1 receives direct dopaminergic inputs from the locus coeruleus^63^ as well as from the ventral tegmental area (VTA). The LECs also receives dopaminergic inputs from the VTA^36^. Moreover, CA1 receives inputs from the mPFC, via the nucleus reuniens of the thalamus, which are crucial for representing the future route during goal-directed behavior^64^. This mPFC input to the CA1 may have contributed to the action-related activities of CA1 neurons, which preceded those of LEC neurons. Intrahippocampal circuits, particularly recurrent circuits in CA3 regions, may also play a role in amplifying event information from the LEC and thus leading to the sharp activity peak observed in CA1.

CA1 represented both the internal (voluntary action) and external (reward) events in contiguity with the actual timings of events, i.e., in real time whereas the LEC showed delayed representation of the same events. When an animal is in a certain place at a particular time, place cells and time cells are activated in CA1. In addition, if a certain event occurs, CA1 neurons immediately respond to that event, resulting in representation of the event as it occurs in real time. Thus, CA1 could represent the specific event by means of a snapshot of specific spatiotemporal information. In contrast, the EC more universally represents spatiotemporal information^13–15,22,25^ in an ongoing manner, possibly like a movie^23^. Triggered by the event, CA1 takes the snapshot from the entorhinal movie, and the EC processes the hippocampal snapshot in the space of universal information before transferring it to the mPFC as the central executive system. In this way, animals can use ongoing information to optimize their behaviors through the entorhinal-hippocampal circuit.

## Methods

### Animals

All experiments were approved by the Animal Research Ethics Committee of Tamagawa University (animal experiment protocol, H22/27-32), and were carried out in accordance with the Fundamental Guidelines for Proper Conduct of Animal Experiment and Related Activities in Academic Research Institutions (Ministry of Education, Culture, Sports, Science, and Technology of Japan) and the Guidelines for Animal Experimentation in Neuroscience (Japan Neuroscience Society). All surgical procedures were performed under appropriate isoflurane anesthesia (see below). All effort was made to minimize suffering. The procedures for our animal experiments were established in our previous studies^65–67^. This study is based on data from channelrhodopsin-2 (ChR2)–expressing (Thy1-ChR2) transgenic rats (W-TChR2V4; *N* = 25 rats, male, 316 ± 39 g, >3 months) abundantly expressing ChR2-Venus fusion protein under the control of the *Thy1.2* promoter in cortical and other neurons^52,68^. These animals were kept in their home cage under an inverted light schedule (lights off at 9 a.m., lights on at 9 p.m.).

### Surgery

Rats were handled briefly by the experimenter (10 min, twice) before the day of surgery. For head plate implantation, rats were anesthetized with isoflurane (4.5% for induction and 2.0–2.5% for maintenance; Pfizer Japan Inc., Tokyo, Japan) using an inhalation anesthesia apparatus (Univentor 400 anesthesia unit, Univentor, Zejtun, Malta) and placed on a stereotaxic frame (SR-10R-HT, Narishige, Tokyo, Japan). In addition, lidocaine jelly (AstraZeneca, Osaka, Japan) was administered around surgical incisions for local anesthesia. During anesthesia, body temperature was maintained at 37 °C using an animal warmer (BWT-100, Bio Research Center, Tokyo, Japan). The head plate (CFR-2, Narishige) was attached to the skull with small anchor screws and two combination of dental resin cements (Super-Bond C&B, Sun Medical, Shiga, Japan; Unifast II, GC Corporation, Tokyo, Japan). Reference and ground electrodes (Teflon-coated silver wires, A-M Systems, Sequim, WA, USA; 125 µm in diameter) were implanted above the cerebellum. Analgesics and antibiotics were applied after the operation (meloxicam, 1 mg/kg s.c., Boehringer Ingelheim Japan, Tokyo, Japan; gentamicin ointment, 0.1% ad usum externum, MSD, Tokyo, Japan).

Water deprivation was started after full recovery from surgery (6 d postoperatively). The rats had *ad libitum* access to water during weekends, but during the rest of the week they obtained water only by performing the task correctly. When necessary, an agar block (containing 15 ml water) was given to the rats in their home cage to maintain them at >85% of their original body weight^66,69,70^.

### Behavioral task

We used the self-paced, spontaneous, left or right pedal-releasing task in our original system (custom made by O’HARA & Co., Ltd., Tokyo, Japan; Fig. 1a; see also refs. ^45–47^) to examine the timing of neural representation of two distinct behavioral events related to learning in CA1 and the LEC. In this task, the rats had to manipulate left and right pedals with the corresponding forelimb in a head-fixed condition. They spontaneously started each trial by pushing both pedals down with both forelimbs and holding them down for a short period (“holding period,” at least 1 s; Fig. 1a). After completing the holding period, the rats had to release either the left or the right pedal, depending on the context without any instruction cue, to obtain 0.1% saccharin water (10 μl) as a reward. The reward was dispensed from the tip of a spout by a micropump with a 300–700 ms delay (100 ms steps at random, Fig. 1a). This task consisted of two blocks, right pedal-rewarded and left pedal-rewarded blocks. Each block lasted until the rat performed more than 30 correct (rewarded) trials and achieved 80% correct performance in the 10 most recent trials or until 100 rewards had been obtained. If the rats incorrectly released the other pedal (error trial) or failed to complete the holding period (immature trial), then they did not receive feedback. The rats typically learned this task within 2 weeks (2–3 h per day).

After 2 days of habituation in the experimental setup (2 h/day), rats in the pre-training group underwent a second surgery under isoflurane anesthesia for later recording experiments. As for the post-training group, once the rats completed task learning, they underwent a second surgery under isoflurane anesthesia for later recording experiments. We made tiny holes (1.0–1.5 mm in diameter) in the skull and dura mater above CA1 (3.0 and 4.5 mm posterior, 2.0 mm lateral from bregma), the LEC (6.0 mm posterior, 6.8 mm lateral), and the mPFC (3.5 mm anterior, 0.6 mm lateral). LEC and CA1 coordinates were determined in our previous study^11,71^. Craniotomy to access the primary motor cortex (M1) was performed at the following coordinate: 1.0 mm anterior, ±2.5 mm lateral^45–47^. All holes were immediately covered with silicon sealant (DentSilicone-V, Shofu, Kyoto, Japan) until the recording experiments.

### In vivo electrophysiological recording

We performed extracellular multi-neuronal (multiple, isolated, single-unit) recordings from individual neurons while the rats were performing behavioral tasks. A supportive layer of agarose gel (2% agarose-HGT, Nacalai Tesque, Kyoto, Japan) was placed on the brain, and then 32-channel silicon probes (Iso_3x_tet-A32 or Iso_4x_tet-A32; NeuroNexus Technologies, Ann Arbor, MI, USA) were precisely inserted into CA1 and the LEC. Insertions were performed using fine micromanipulators (SM-15 or SMM-200B, Narishige) at least 1 h before the start of each recording experiment.

Wide-band signals were amplified and filtered (FA64I, Multi Channel Systems, Reutlingen, Germany; final gain, 2000; band-pass filter, 0.5 Hz to 10 kHz) through a 32-channel head stage (MPA32I, Multi Channel Systems; gain, 10). These signals were digitized at 20 kHz and recorded with three 32-channel hard-disc recorders (LX-120, TEAC, Tokyo, Japan) that simultaneously digitized the pedal positions tracked by angle encoders and the events resulting from optogenetic stimulation.

### Optogenetic stimulation

We used the Multi-Linc method to effectively identify pyramidal neurons sending direct projections to specific areas by combining multi-areal optogenetic stimulation and multi-neuronal recordings. Details of this procedure were described previously^52^. Briefly, prior to the insertion of silicon probes, the optical fibers (FT400EMT, FC, NA, 0.39; internal/external diameters, 400/425 μm; Thorlabs, Newton, NJ, USA) for stimulation were vertically inserted into the mPFC (4100 μm deep) and CA1 (2300 μm deep) using micromanipulators (SM-25A, Narishige). To evoke antidromic spikes in specific axonal projections from LEC neurons (mPFC- and CA1-projecting cells), a blue LED light pulse (intensity, 5–10 mW; duration, 0.5–2 ms, typically 1 ms) was applied through each of the two optical fibers using an ultra-high-power LED light source (UHP-Mic-LED-460, FC, Prizmatix Ltd., Givat-Shmuel, Israel) and a stimulator (SEN-8203, Nihon Kohden, Tokyo, Japan). To be classified as projecting neurons, neurons were required to meet several criteria, including constant latency, fixed frequency (frequency-following test, two pulses at 100 and 200 Hz), and collision test^45,46,50–52,59,72^.

### Spike isolation

Raw signal data were processed offline to isolate spike events of individual neurons in each tetrode of the silicon probes. Briefly, spike candidates were detected and clustered by our semiautomatic spike-sorting software, EToS^73,74^. Using open source software (Klusters clustering software and NeuroScope viewing software^75^) spike clusters were manually combined, divided, discarded, or subjected to a combination thereof to refine single-neuron clusters based on two criteria: the presence of refractory periods (>2 ms) in their own autocorrelograms and the absence of refractory periods in cross-correlograms with other clusters. We included single-neuron clusters if they exhibited a sufficient number of spike trains during task performance (≥20 trials with total ≥250 spikes). These clusters were classified as either task-related or non-task-related neurons (Fig. 2e and Supplementary Fig. 4; see also below).

### Spike collision analysis

To identify mPFC- and CA1-projecting LEC neurons, we used the Multi-Linc method with post hoc analysis to complete multi-neuronal collision tests^52^. Briefly, after offline sorting for spike isolation, we used MATLAB (MathWorks, Natick, MA, USA) to compare filtered traces with no spikes prior to the stimulus (control traces, colored black in Fig. 1f) against those that had a spike in one spike cluster (test traces, colored red in Fig. 1f). If we found antidromic-like spike activities (all-or-none and no jittering; black arrowheads in Fig. 1f) with short latency in many of the control traces, we set a time window for counting possible antidromic spikes, based on a clear dissociation between averaged control and test traces due to the presence or absence of spikes. The cut-off threshold defined in a receiver operating characteristic curve for distributing the most negative points (trough of spike waveform) within the time window was used to determine whether spikes were present, so that we obtained spike and no-spike counts in the control and test events. Based on this method, we included spike clusters with a control spike probability above 50% and a test spike probability that was less than half the value of the control. Finally, to determine statistically if the collision test was passed, we performed a 2 × 2 χ^2^ test (*p* < 0.05) of spike and no-spike counts in control and test events (see Supplementary Fig. S9 in ref. ^52^). The latency of antidromic spikes was defined as the time from the onset of stimulation to the median of the peak spike positions within the time window, and their jitter was defined as the time between the first (25%) and the third (75%) quartiles of their peak positions within the time window. In this way, we judged if these spikes were antidromic based on the collisional disappearance of antidromic spikes (collision test), as well as their all-or-none properties, absence of jitter (constant latency test; <0.5 ms), and high reliability (frequency-following test; if applicable in the tentative collision test).

### Spike analysis

Within each neuron (spike cluster), basal spiking properties and task-related activity in relation to behavioral task performance were analyzed using MATLAB as follows. The ongoing spike rate and spike duration (onset to peak) for individual spike clusters were defined as in our previous studies^46,52,76^. Spike clusters were classified as RS neurons (mostly putative excitatory) and FS neurons (putative inhibitory) based on spike duration, with the clear bimodal distribution of spike duration divided into two clusters by minimum cross entropy thresholding of spike duration^77,78^ (≥0.72 ms for RS neurons, <0.72 ms for FS neurons; Supplementary Fig. 3). Since we refer to many groups of neurons (e.g., RS vs. FS, CA1 vs. LEC), we use abbreviations for simplicity e.g., CA1-RS for RS neurons in CA1.

Next, we examined task-related spike activity correlated with self-initiated action or outcome (reward delivery). For action-related activity, we analyzed spike trains in relation to unilateral forelimb movements during task performance; these spike trains were aligned with the onset (0 s) of pedal release (following ≥1 s holding time, window: onset ±500 ms). The range of motion for the pedal was 0–100% and the holding area was defined as 0–30% (Fig. 1a). For task progression, pedal release was detected as the time at which the pedal moved beyond the holding area. In order to more precisely detect the release onset for neuronal analysis, pedal release was defined as the time when the pedal position exceeded 5% in the pedal position before approximate pedal release (detection by 30%, see above). The task-related activity was defined by the task relevance index using the Kolmogorov–Smirnov (KS) test, as previously described^52,79^ (Supplementary Fig. 4a; see also Fig. 2a in ref. ^46^); Briefly, the cumulative distribution of all spike positions in the time course of each trial was compared with that of the same number of uniformly distributed spike positions, where a task-related neuron was defined as a neuron with a task relevance index (*p* value of KS test) smaller than the criterion (*p* = 10^−6^) in contra- or ipsilateral pedal release trials. The preference activity (contra- or ipsilateral) was defined as the side with the smaller task relevance index. Task-related neurons were further classified into Hold-type and Go-type according to the peak time position of spike increase and the dependence on pedal holding time in the PETH (20 ms bins) on the preferred side^45–47^. A Hold-type neuron has a sustained spike increase prior to the release onset (0 s), and this increase depends on the holding time. A Go-type neuron has a phasic spike increase that is independent of the holding time.

To check the limb preference of Go-type neurons, we compared the peak amplitude of contralateral and ipsilateral release trials. Peak amplitude was calculated by averaging the spike rate in the peak period (center of peak bin ±150 ms), in which the peak bin was determined in the PETH of preferred release trials (contralateral or ipsilateral). We used the same peak period to calculate peak amplitude in non-preferred release trials. For Hold-type neurons, we compared the mean spike rate during the holding period (−1000 to 0 ms) between contra- and ipsilateral release trials. Moreover, we evaluated the limb preference (laterality) of Go-type neurons using the laterality index (ranging from −1 to +1)^45–47^ based on normalized peak activities as follows,

$${Laterality}\,{index}=\left\{\begin{array}{cc}(c-i)/(c+i), & {if}\,c \, > \, 0\,{and}\,i \, > \, 0\\ +1, \hfill& {if}\,c \, > \, 0\,{and}\,i \, < \, 0\\ -1, \hfill& {if}\,c \, < \, 0\,{and}\,i \, > \, 0\end{array}\right.$$

where *c* and *i* are activities associated with contralateral and ipsilateral movements, respectively. These parameters were obtained from the following equation:

$$c,\,i={{SR}}_{{peak}}/{{SR}}_{{baseline}}-1$$

where *SR*_*peak*_ is the mean spike rate in the peak period (center of peak bin ±150 ms), and *SR*_*baseline*_ is the mean spike rate in the baseline period (−1000 to −700 ms relative to pedal release onset). Consequently, laterality index values of −1 and +1 indicate ipsilateral- and contralateral-preferring neuronal activity, respectively.

For outcome-related activity, spike trains were aligned with the onset (0 s) of reward delivery (window: onset to 1000 ms), and outcome-related activity (Reward-type) was defined in the same way as action-related activity (Supplementary Fig. 4a). When neurons were classified as being related to both action and outcome based on PETHs aligned with pedal release and reward delivery, they were called action- and outcome-related types, e.g., Go&Reward-type or Hold&Reward-type, according to the action-related activity.

The reward modulation index for go- and reward-related activity was calculated from the following equation^46^:

$${Reward}\,{modulation}\,{index}=({{SR}}_{r}-{{SR}}_{{nr}})/({{SR}}_{r}+{{SR}}_{{nr}})$$

where *SR*_*r*_ and *SR*_*nr*_ are the mean spike rates during the peak period (go-related: peak ±250 ms; reward-related: reward onset to 500 ms) for rewarded (correct) and non-rewarded (error) trials, respectively. If this index is >0, the activity is considered positively modulated by reward.

### Generalized linear regression model

We used a generalized linear regression model to test each cell individually. We fitted this model using the MATLAB stepwiseglm function to determine how spike discharges of individual neurons were related to the following variables: right pedal trajectory, left pedal trajectory, and trial time. Our task did not have an instruction cue so we defined the trial time as the duration between the time that a rat pushed both pedals down to the next instance of the same rat pushing both pedals down (Fig. 4b). Terms were added or removed from the model when the deviation by these operations was significantly large (*F*-test or chi-squared test, *p* = 0.05 for adding and = 0.10 for removing). The smoothed spike rate (σ = 150 ms for spikes in 0.05-ms bins) was estimated for each session. The MATLAB stepwiseglm function also returns *p*-values of each predictor that significantly predicts the PETH in the generalized linear regression model. These *p*-values with Bonferroni correction were used to classify cells as Time-, Time&Pedal-, and Pedal-correlated cells.

### GMM with BIC

Peak latencies extracted from PETHs of spike activity associated with pedal release and reward delivery were clustered into several groups based on the assumption of the GMM. We assumed that a distribution of the peak latencies could be represented with a small number of Gaussian distributions. Basically, the number of Gaussian distributions should be set by a user in advance as a hyperparameter. We repeated the GMM fitting with the expectation-maximization (EM) algorithm 1000 times for cluster numbers of 1–5, and calculated a BIC for each repeat:

$${BIC}=-2\, {{{{{\rm{ln}}}}}}\left(L\right)+k{{{{{\rm{ln}}}}}}(n)$$

where *L*, *k*, and *n* indicate the likelihood of each sample, number of parameters, and number of samples, respectively. Then we defined the optimal cluster number that showed the minimum mean BIC (Supplementary Fig. 7a). We also used the *x*-means clustering algorithm^80^ (Supplementary Fig. 7b), which is an extension of the *k*-means clustering algorithm, to re-confirm clustering results with an algorithm other than the GMM clustering algorithm.

To calculate pseudo-paired differences in peak latencies between neurons in three different areas, we conducted a 1000-repeat bootstrap analysis. This pseudo-signal flow analysis was conducted by using pooled data across all animals (colored dots in Fig. 5f; differences in latencies between simultaneously recorded neurons in a representative animal are shown by black dots). Each step of the bootstrap selects one neuron from each region to calculate inter-region peak time lags. We clustered the differences in time lags into several groups using two-dimensional GMM fitting (i.e., CA1 vs. LECs and CA1 vs. LECd). The optimal number of clusters was defined by the BIC (Supplementary Fig. 7c). These procedures were performed with custom scripts written in Python (ver. 3.9; Python Software Foundation, DE, US) along with some additional modules such as scikit-learn^81^ (for 1D and 2D GMM fittings with the EM algorithm following BIC calculation) and PyClustering^82^ (for *x*-means clustering).

### Histological observations

After the recording experiments, animals were deeply anesthetized with urethane (2–3 g/kg, i.p., Nacalai Tesque) and transcardially perfused with cold saline followed by 4% formaldehyde in 0.1 M phosphate buffer. Whole brains were postfixed and sliced coronally into 50-μm serial sections using a microslicer (VT1000S, Leica, Wetzlar, Germany). Electrode tracks labeled with 1,1’-dioctadecyl-3,3,3’,3’-tetramethylindocarbocyanine perchlorate (DiI, Thermo Fisher Scientific, Waltham, MA, USA) were observed in CA1 and the LEC under a fluorescence microscope (BX51N, Olympus, Tokyo, Japan).

### Retrograde tracing

Rats were anesthetized with isoflurane in an induction chamber and then moved to an inhaling mask on a stereotactic frame. The skull was exposed and a small burr hole was drilled above the injection site. The injection was made by means of a glass micropipette (tip diameter = 20–40 µm) connected to a 1-µL Hamilton microsyringe. Rats received 100 nL of Fluoro-Gold (2.5% in H_2_O, Fluorochrome) and 1200 nL of mRFP-expressing G-deleted rabies viral vector (rHEP5.0-ΔG-mRFP; 6.0 ×10^8^ focus-forming units/mL)^49^ into the mPFC (AP = +3.5 mm; ML = 0.6 mm; DV = −2.6 mm) and the dorsal hippocampus (AP = −4.4 mm; ML = 1.8 mm; DV = −2.6 mm), respectively. Injection site coordinates were based on the rat brain atlas^83^ and calculated from bregma. Seven days into the survival period, rats were deeply anesthetized with sodium pentobarbital (100 mg/kg, i.p.) and perfused transcardially with Ringer’s solution (0.85% NaCl, 0.025% KCl, 0.02% NaHCO_3_) followed by 4% paraformaldehyde in 0.1 M phosphate buffer. Brains were removed from skulls, postfixed in 4% paraformaldehyde in 0.1 M phosphate buffer for 4 h at 4 °C, and cryoprotected in a mixture of 20% glycerol and 2% dimethyl sulfoxide. The brains were cut into 40-µm sections in the coronal plane on a freezing microtome. Sections were counterstained with mouse anti-NeuN antibody (Millipore Burlington, MA, USA, #MAB377) as described previously^10^, mounted on gelatin-coated slides, and covered with Entellan new (Millipore, #107961) before a coverslip was applied. Axio Scan. Z1 (Carl Zeiss, Oberkochen, Germany) and ZEN 2 software (Carl Zeiss) were used to image labeled neurons.

### Experimental design

We obtained electrophysiological data from 25 sessions in 25 Thy1-ChR2 rats (pre-training, *N* = 11, 65.2 ± 49.2 cells/rat; post-training, *N* = 14, 105.0 ± 58.2 cells/rat) to examine behavioral event representations of the CA1 and LEC neurons. This “one rat, one recording” approach enabled us to accurately reconstruct the probe position, which was important to distinguish the LEC layers (Fig. 1). In total, we included data from 829 CA1 neurons (pre-training, 296 cells; post-training, 533 cells), and 1287 LEC neurons (pre-training, 370 cells; post-training, 917 cells) during task performance (see Results for details). These neurons were divided into RS and FS subclasses by spike duration, and further classified into Go-type, Go&Reward-type, Hold-type, Hold&Reward-type, and Reward-type neurons if they were functionally related to task events (Figs. 2–5). Because there was no significant correlation between the fraction of task-related cells and rats’ performances after training (fraction: 24.7 ± 7.1%, correct rate: 82.9 ± 6.7%, Pearson correlation coefficient*, r* = 0.23, *p* = 0.44), we pooled the data obtained from post-trained rats for analysis. We also used data from our previous study (M1 Hold-type neurons, 43 cells from 12 rats)^46,47^.

### Statistics and reproducibility

Standard replication of measurements were performed for this study. The reported findings were reproduced across animals. All quantifications were conducted at the single-neuron level. Sample sizes (the numbers of animals, sessions, and neurons) were estimated according to previous studies^45–47^ and confirmed to be adequate by power analyses (power = 0.9; alpha error = 0.05). We used the following statistical methods: KS test, Mann–Whitney test, one-sample signed-rank test, Wilcoxon signed-rank test, χ^2^ test with post hoc residual analysis, and Kruskal–Wallis test with post hoc Steel–Dwass test. All tests were two-sided unless otherwise stated. These statistical tests were conducted with MATLAB’s Statistics and Machine Learning Toolbox (MathWorks). Differences were considered statistically significant when *p* < 0.05 (see Results for details). Blinding and randomization were not performed.

### Reporting summary

Further information on research design is available in the Nature Portfolio Reporting Summary linked to this article.

## Supplementary information

Supplementary Information

Description of Additional Supplementary Files

Supplementary Data 1

Reporting Summary

## Data Availability

All source data for graphs can be found in Supplementary Data 1. Other data are available from the corresponding author on reasonable request.

## Code availability

All original code generated in this study is available upon request.
