# Supplementary material for: Rat hippocampal CA1 region represents learning-related action and reward events with shorter latency than the lateral entorhinal cortex
Source: Commun Biol. 2023 May 31;6:584. doi: 10.1038/s42003-023-04958-0 (PMC10232461; doi:10.1038/s42003-023-04958-0)
Supplement: Supplementary file 1 — Supplementary Information [file 42003_2023_4958_MOESM1_ESM.pdf]

Supplementary Information for

Rat hippocampal CA1 region represents learning-related action and reward events with shorter latency than the lateral entorhinal cortex

Shogo Soma[1,2,†,*], Shinya Ohara[3,4,†], Satoshi Nonomura[1,5,6], Naofumi Suematsu[7], Junichi Yoshida[1,8], Eva Pastalkova[9], Yutaka Sakai[1], Ken-Ichiro Tsutsui[3], and Yoshikazu Isomura[1,5,*]

[1]*Brain Science Institute, Tamagawa University, Tokyo, Japan.* [2]*Department of Molecular Cell Physiology, Kyoto Prefectural University of Medicine, Kyoto, Japan.* [3]*Laboratory of Systems Neuroscience, Tohoku University Graduate School of Life Sciences, Sendai, Japan.* [4]*PRESTO, Japan Science and Technology Agency (JST), Kawaguchi, Japan.* [5]*Department of Physiology and Cell Biology, Graduate School of Medical and Dental Sciences, Tokyo Medical and Dental University, Tokyo, Japan.* [6]*Center for the Evolutionary Origins of Human Behavior, Kyoto University, Aichi, Japan.* [7]*Department of Bioengineering, University of Pittsburgh. Pittsburgh, PA, USA.* [8]*Dominick P. Purpura Department of Neuroscience, Albert Einstein College of Medicine, Bronx, NY, USA.* [9]*Department of Clinical Psychology, Pacifica Graduate Institute, Carpinteria, CA, USA.* [†]These authors contributed equally.

*Shogo Soma and Yoshikazu Isomura
E-mail: soma@koto.kpu-m.ac.jp (lead contact), isomura.phy2@tmd.ac.jp

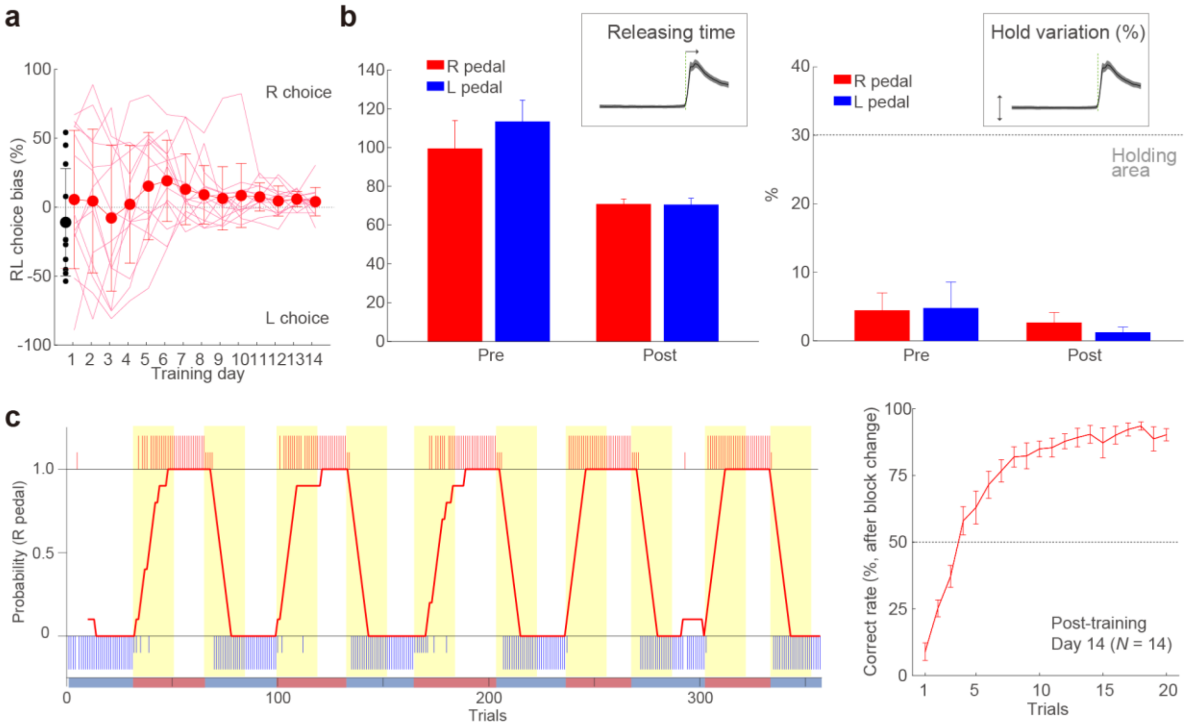

Supplementary Figure 1. Behavioral task performance on the self-paced, spontaneous left or right pedal-releasing task. a Left-right choice bias (RL choice bias). Both groups were able to manipulate the individual pedals spontaneously. The preference for one pedal over the other decreased during training. **b** The stability of the holding and releasing time (time from the onset to end of release) were developed over the training. The rats of the post-training group quickly released the pedal (releasing time [ms], group, $F_{(1,49)} = 17.9$, $p < 1.1 \times 10^{-4}$, $\eta G^2 = 0.27$; right vs left, $F_{(1,49)} = 0.50$, $p = 0.48$, $\eta G^2 = 0.01$) and returned their forelimb to the pedal after stable pedal holding (Holding variation was defined as SD of pedal trajectory, two-way ANOVA, group, $F_{(1,49)} = 16.1$, $p < 2.1 \times 10^{-4}$, $\eta G^2 = 0.25$; right vs left, $F_{(1,49)} = 0.97$, $p = 0.33$, $\eta G^2 = 0.02$). **c** Example of task performance (left). Highlight indicates the trials for calculation of correct rate after the block change (right). Averaged proportion of correct choices in the first to 20th trials after the change of blocks. The number of trials to achieve the reversal (>50%) of the proportion of correct choices after the block change is 4.5 [4, 6] trials (median [IQR]).

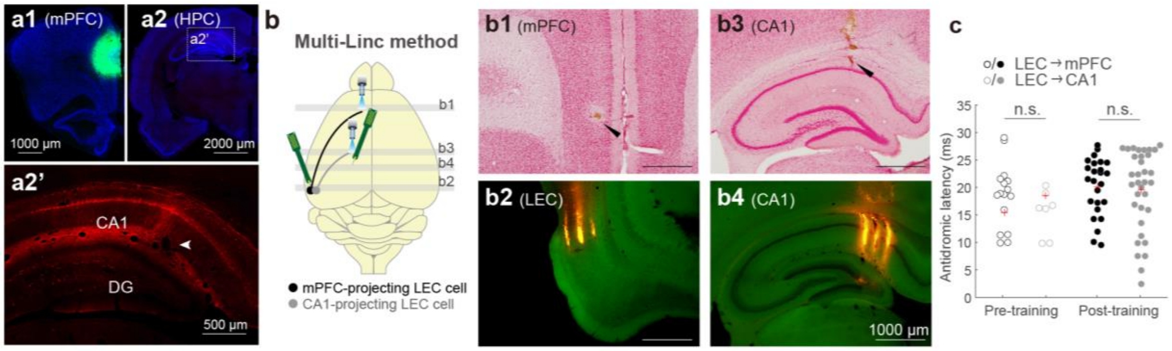

Supplementary Figure 2. Effective LEC recording method. a LEC neurons projecting to mPFC and hippocampus. Retrograde tracer (Fluoro-Gold, green) was injected into the mPFC (**a1**), while retrograde viral tracer (mRFP-expressing G-deleted rabies viral vector, red) was injected into the hippocampus (**a2**). The distribution of retrogradely labeled neurons was examined in the LEC (Fig. 1e). Blue, NeuN. **b** Schema showing the position of optical fibers for identifying the two different projection neurons in the LEC (left). The ipsilateral mPFC and CA1 were stimulated to identify mPFC- and CA1-projecting LEC neurons, respectively. Images on the top right show the stimulation sites in the mPFC (**b1**) and CA1 (**b3**). Arrowheads show tracks of the optical fibers into the mPFC and CA1 in Nissl-stained sections. Images on the bottom right show recording sites in the LEC (**b2**) and CA1 (**b4**). Probe shank tracks were visualized with fluorescent DiI (red). Scale bars, 1,000 µm. **c** Spike latency after antidromic stimulation in mPFC- (black) and CA1-projecting (gray) LEC neurons (right).

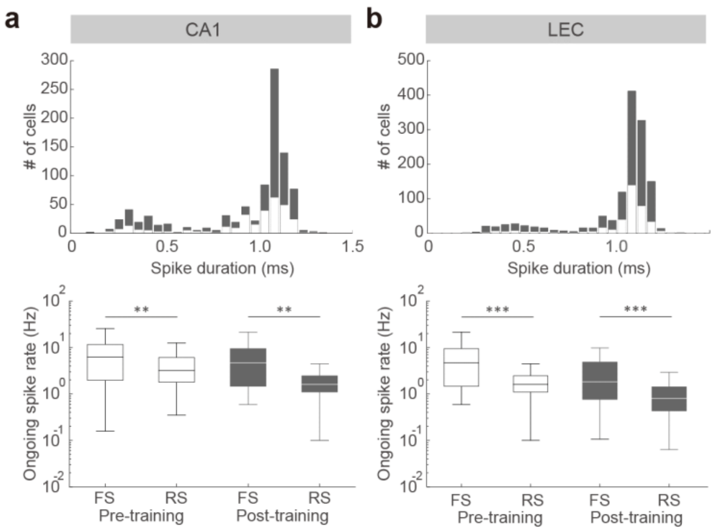

Supplementary Figure 3. Classification of RS and FS neurons in CA1 and the LEC. a-b Recorded neurons were divided into RS and FS neurons based on spike duration (RS: ≥0.72 ms, FS: <0.72 ms). This threshold was determined by minimum cross-entropy thresholding[77,78], which derives the optimal value for classifying the distribution into two categories based on the histogram. The spike duration was defined as the time from spike onset to the first positive peak. Top, bimodal distribution of spike durations. White and gray colors represent neurons obtained from pre- and post-training groups, respectively. Bottom, box plot shows ongoing (all averaged) spike rates (middle bar, median; upper and lower edges, quartiles; whiskers, error) for FS and RS neurons in CA1 (**a**) and the LEC (**b**). The ongoing spike rates of FS neurons were significantly higher than those of RS neurons in CA1 and the LEC (pre-training: CA1-RS, n = 247, median [IQR] in Hz, 3.2 [1.8, 6.1], CA1-FS, n = 49, 6.2 [2.0, 11,5]; Mann–Whitney test, $z = -2.97$, $p < 3.0 \times 10^{-3}$, $r = 0.17$; LEC-RS, n = 335, 1.6 [1.1, 2.4], LEC-FS, n = 35, 4.7 [1.5, 9.4]; $z = -4.60$, $p < 4.2 \times 10^{-6}$, $r = 0.24$; post-training: CA1-RS, n = 433, 2.8 [1.1, 5.3], CA1-FS, n = 100, 3.8 [1.5, 8.8]; $z = -2.98$, $p < 2.9 \times 10^{-3}$, $r = 0.12$; LEC-RS, n = 789, 0.8 [0.4, 1,4], LEC-FS, n = 128, 1.8 [0.8 ,4.9]; $z = -7.38$, $p < 1.5 \times 10^{-13}$, $r = 0.24$). We also found a significant reduction in ongoing spike rates in FS neurons after learning (CA1: $z = -2.90$, $p < 3.8 \times 10^{-3}$, $r = 0.22$; LEC, $z = -3.0$, $p < 2.8 \times 10^{-3}$, $r = 0.23$; see the main text for RS neurons). ** $p < 0.01$, *** $p < 0.001$, Mann–Whitney test.

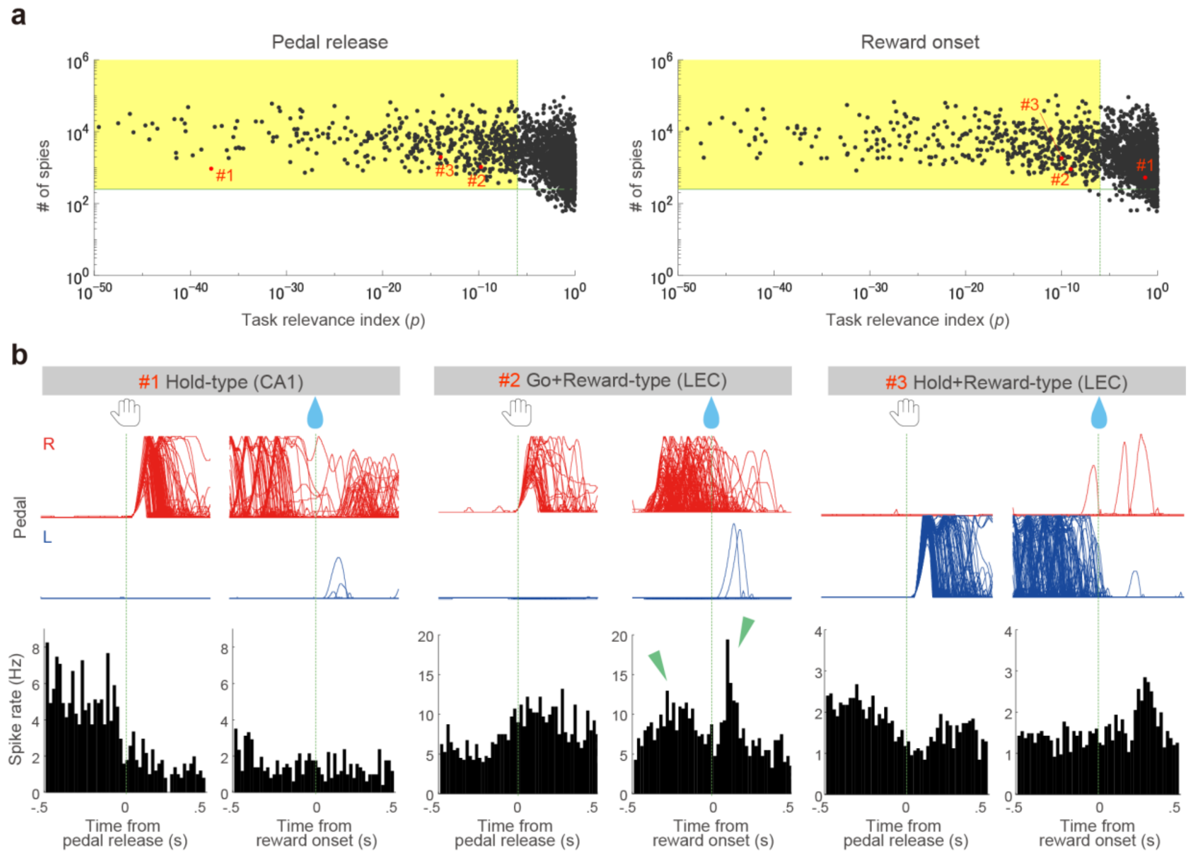

Supplementary Figure 4. Definitions of task-related neurons and examples of Hold-type, Go&Reward-type, and Hold&Reward-type task-related activities in CA1 and the LEC after learning. a The number of spikes during pedal release and reward delivery are plotted against the task relevance index for individual neurons (see Methods). A pale yellow background indicates the area in which activities were defined as task related (green line, $p < 10^{-6}$; green dashed line, ≥250 spikes) Red numbers correspond to examples of activities (**b**). **b** Spikes are aligned with the onset of pedal release (left column) or reward delivery (right column) at 0 s for each cell. The Go&Reward-type neuron shows increased activity in both the pedal-release and reward-delivery periods (two peaks indicated by green arrowheads). Top, pedal trajectories (R: right pedal, L: left pedal). Bottom, PETHs (bin width, 20 ms).

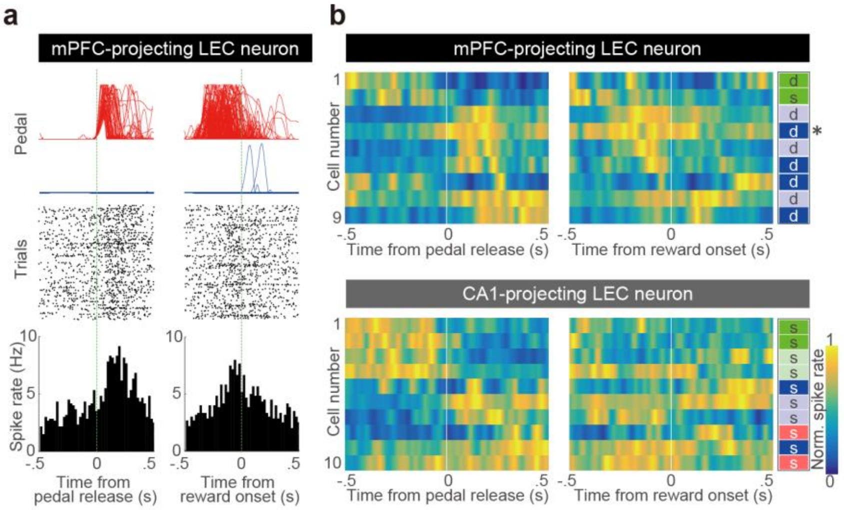

Supplementary Figure 5. Task-related activity of mPFC- and CA1-projecting neurons. a Examples of Go-type task-related activity in the LEC. The Multi-Linc method identified this neuron as projecting to the mPFC (see Methods). Top, middle, and bottom show pedal trajectories (red: right pedal, blue: left pedal), spike raster plots, and PETHs (bin width, 20 ms), respectively. Spike data were aligned with the pedal-release onset (left) or reward onset (right) at 0 s for individual task-related neurons. **b** Task-related activity of mPFC- (top) and CA1-projecting (bottom) neurons in the LEC. The figure legend is the same as Fig. 3a. The task-related type is indicated on the right side with layer position (s: superficial layer, d: deep layer). * represents the example neuron shown in **a**.

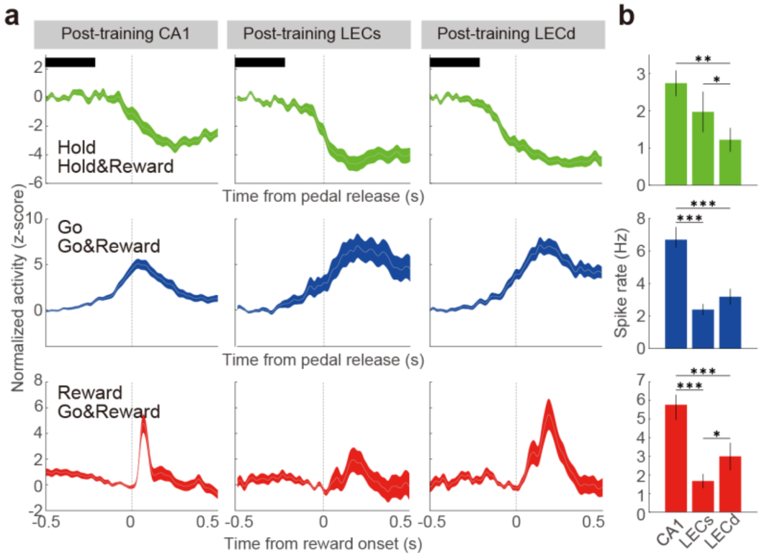

Supplementary Figure 6. Differences in task-related activity between CA1, LECs, and LECd. **a** Averaged PETHs of all hold-related (Hold-type and Hold&Reward-type), go-related (Go-type and Go&Reward-type), and reward-related (Reward-type and Go&Reward-type) activities in CA1 (left), LECs (middle), and LECd (right). PETHs were aligned with pedal-release onset (top and middle) and reward onset at 0 s (bottom). Shaded regions represent 95% CIs. **b** Comparison of spiking activities between CA1, LECs, and LECd. CA1 neurons show greater activity than LEC neurons. Horizontal black bars in the hold-related activity (**a**) indicate the windows for calculating the spike rate. The spike rate for go- and reward-related activities were calculated from the peak period (peak ± 150 ms). In the hold-related activity, LECs neurons show a higher spike rate than LECd neurons. In contrast, LECd neurons show a higher spike rate than LECs neurons in the reward-related activity. * $p < 0.05$, ** $p < 0.01$, *** $p < 0.001$, post hoc Steel–Dwass test.

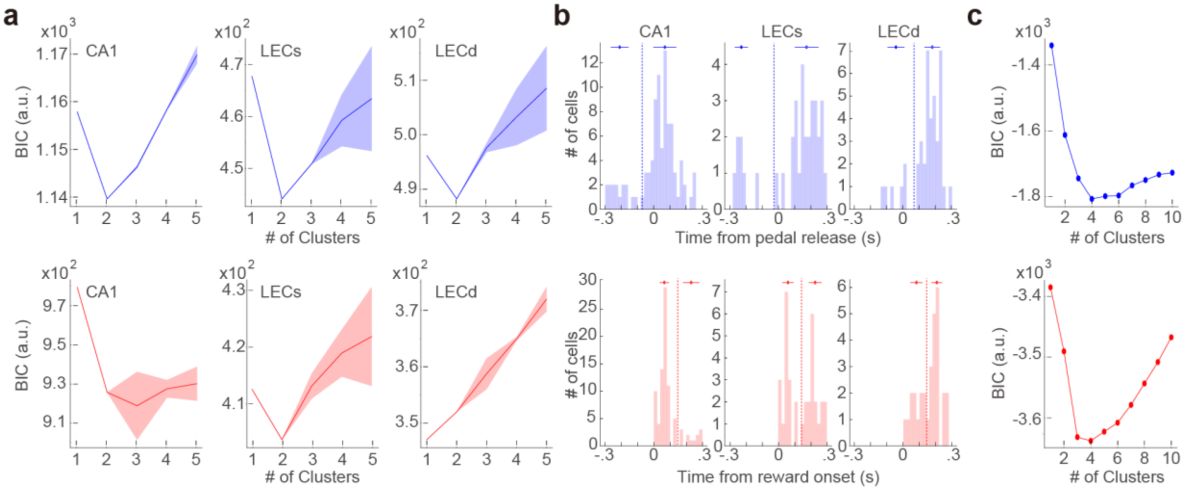

Supplementary Figure 7. BIC values and peak latency clustering with different methods. a BIC values for peak latency distributions for cluster numbers 1 to 5 (Fig. 5e). Mean ± SD. Fitting with one or two clusters resulted in the minimum BIC. **b** *x*-means clustering of peak latencies in CA1 (left), LECs (middle), and LECd (right), aligned with pedal-release (top) and reward-onset (bottom) timings. Symbols and horizontal lines indicate mean and ± SD of each cluster, respectively. Dotted vertical lines are cluster boundaries. **c** BIC values for the pseudo-paired latency difference distributions for cluster numbers 1 to 10 (Fig. 5f). Fitting with four clusters resulted in the minimum BIC for both pedal-release and reward-onset alignment conditions.

Supplementary Table 1. Comparison of peak activity triggered by pedal release in contra- and ipsilateral trials (Mann–Whitney test)

Area	Activity type	Contra (Hz)	Ipsi (Hz)	*z* value	*p* value	Effect size (*r*)
CA1	Go-type	5.6 [3.3,8.0]	6.6 [3.9,8.9]	-3.07	2.1×10^{-3}	0.30
	Go&Reward-type	7.6 [4.1,13.8]	9.0 [5.0,12.8]	-0.83	0.40	0.10
LECs	Go-type	1.2 [0.7,3.4]	1.6 [1.0,2.7]	0.70	0.48	0.10
	Go&Reward-type	2.3 [1.1,4.2]	1.6 [1.1,4.6]	-1.10	0.30	0.10
LECd	Go-type	2.5 [1.4,4.0]	2.0 [0.9,3.3]	1.53	0.13	0.20
	Go&Reward-type	3.4 [1.2,7.0]	4.0 [2.3,7.0]	-0.73	0.50	0.10

Values are median [IQR]

Supplementary Table 2. Comparison of peak activity triggered by reward onset in contra- and ipsilateral trials (Mann–Whitney test)

Area	Activity type	Contra (Hz)	Ipsi (Hz)	*z* value	*p* value	Effect size (*r*)
CA1	Reward-type	3.5 [1.9,7.0]	3.6 [1.7,7.0]	-1.73	0.08	0.18
	Go&Reward-type	7.5 [3.9,13.8]	8.9 [3.6,12.8]	-0.70	0.48	0.08
LECs	Reward-type	1.1 [0.7,1.7]	1.1 [0.7,2.1]	-1.49	0.15	0.08
	Go&Reward-type	2.3 [0.9,4.1]	1.4 [0.8,4.6]	-0.51	0.61	0.07
LECd	Reward-type	1.5 [0.4,2.2]	1.6 [1.1,2.3]	-1.16	0.25	0.20
	Go&Reward-type	2.8 [1.4,6.4]	3.9 [2.0,6.9]	-0.87	0.41	0.08

Values are median [IQR]
