# Supplementary material for: Rat hippocampal CA1 region represents learning-related action and reward events with shorter latency than the lateral entorhinal cortex
Source: Commun Biol. 2023 May 31;6:584. doi: 10.1038/s42003-023-04958-0 (PMC10232461; doi:10.1038/s42003-023-04958-0)
Supplement: Supplementary file 4 — Reporting Summary [file 42003_2023_4958_MOESM4_ESM.pdf]

nature portfolio

Corresponding author(s): Shogo Soma

Last updated by author(s): May 1, 2023

Reporting Summary

Nature Portfolio wishes to improve the reproducibility of the work that we publish. This form provides structure for consistency and transparency in reporting. For further information on Nature Portfolio policies, see our Editorial Policies and the Editorial Policy Checklist.

Statistics

For all statistical analyses, confirm that the following items are present in the figure legend, table legend, main text, or Methods section.

n/a	Confirmed	
☐	☒	The exact sample size (*n*) for each experimental group/condition, given as a discrete number and unit of measurement
☐	☒	A statement on whether measurements were taken from distinct samples or whether the same sample was measured repeatedly
☐	☒	The statistical test(s) used AND whether they are one- or two-sided<br>*Only common tests should be described solely by name; describe more complex techniques in the Methods section.*
☐	☒	A description of all covariates tested
☐	☒	A description of any assumptions or corrections, such as tests of normality and adjustment for multiple comparisons
☐	☒	A full description of the statistical parameters including central tendency (e.g. means) or other basic estimates (e.g. regression coefficient) AND variation (e.g. standard deviation) or associated estimates of uncertainty (e.g. confidence intervals)
☐	☒	For null hypothesis testing, the test statistic (e.g. F, t, r) with confidence intervals, effect sizes, degrees of freedom and P value noted<br>*Give P values as exact values whenever suitable.*
☒	☐	For Bayesian analysis, information on the choice of priors and Markov chain Monte Carlo settings
☒	☐	For hierarchical and complex designs, identification of the appropriate level for tests and full reporting of outcomes
☐	☒	Estimates of effect sizes (e.g. Cohen's *d*, Pearson's *r*), indicating how they were calculated

Our web collection on statistics for biologists contains articles on many of the points above.

Software and code

Policy information about availability of computer code

Data collection	Operant Task StudioV2 (O'hara & Co., Ltd), LX Navi(Version 3.28, TEAC)
Data analysis	EToS (Takekawa et al., 2010, http://etos.sourceforge.net), Klusters(Hazan et al., 2006, http://neurosuite.sourceforge.net/), MATLAB 2017a (Mathworks Inc), Python (ver. 3.9, https://www.python.org/)

For manuscripts utilizing custom algorithms or software that are central to the research but not yet described in published literature, software must be made available to editors and reviewers. We strongly encourage code deposition in a community repository (e.g. GitHub). See the Nature Portfolio guidelines for submitting code & software for further information.

Data

Policy information about availability of data

All manuscripts must include a data availability statement. This statement should provide the following information, where applicable:

- Accession codes, unique identifiers, or web links for publicly available datasets
- A description of any restrictions on data availability
- For clinical datasets or third party data, please ensure that the statement adheres to our policy

All source data for graphs can be found in Supplementary Data 1. Other data are available from the corresponding author on reasonable request.

Human research participants

Policy information about studies involving human research participants and Sex and Gender in Research.

Reporting on sex and gender	N/A
Population characteristics	N/A
Recruitment	N/A
Ethics oversight	N/A

Note that full information on the approval of the study protocol must also be provided in the manuscript.

Field-specific reporting

Please select the one below that is the best fit for your research. If you are not sure, read the appropriate sections before making your selection.

☒ Life sciences ☐ Behavioural & social sciences ☐ Ecological, evolutionary & environmental sciences

For a reference copy of the document with all sections, see nature.com/documents/nr-reporting-summary-flat.pdf

Life sciences study design

All studies must disclose on these points even when the disclosure is negative.

Sample size	No prior calculation of sample size were performed. Sample sizes (the number of animals, sessions, and neurons) were estimated according to previous studies (ref. 45-47).
Data exclusions	All data from animals with silicon probes and optical fibers in targeted areas (CA1 and LEC) were included except for single units that were not stable over the course of recording session.
Replication	Standard replication of measurements were performed for this study. The reported findings were reproduced across animals.
Randomization	The rats were randomly assigned to the pre- and post-training groups after head-plate implantation.
Blinding	Blinding were not performed.

Reporting for specific materials, systems and methods

We require information from authors about some types of materials, experimental systems and methods used in many studies. Here, indicate whether each material, system or method listed is relevant to your study. If you are not sure if a list item applies to your research, read the appropriate section before selecting a response.

Materials & experimental systems

n/a	Involved in the study	
☐	☒	Antibodies
☒	☐	Eukaryotic cell lines
☒	☐	Palaeontology and archaeology
☐	☒	Animals and other organisms
☒	☐	Clinical data
☒	☐	Dual use research of concern

Methods

n/a	Involved in the study	
☒	☐	ChIP-seq
☒	☐	Flow cytometry
☒	☐	MRI-based neuroimaging

Antibodies

Antibodies used	Anti-NeuN (mouse monoclonal, Millipore, MAB377)
Validation	The specificity of primary antibody was validated from manufacture site with references.

Animals and other research organisms

Policy information about studies involving animals; ARRIVE guidelines recommended for reporting animal research, and Sex and Gender in Research

Laboratory animals	Adult W-TChR2V4 rats (ref. 52 and 69) were used for electrophysiology. Young adult Sprague-Dawley rats were used for tracing study.
Wild animals	Not used
Reporting on sex	Findings are not related to the sex of animal. Male rats were only used in this study.
Field-collected samples	Not used
Ethics oversight	All experiments were approved by the Animal Research Ethics Committee of Tamagawa University (animal experiment protocol, H22/27-32), and were carried out in accordance with the Fundamental Guidelines for Proper Conduct of Animal Experiment and Related Activities in Academic Research Institutions (Ministry of Education, Culture, Sports, Science, and Technology of Japan) and the Guidelines for Animal Experimentation in Neuroscience (Japan Neuroscience Society)

Note that full information on the approval of the study protocol must also be provided in the manuscript.
